# Inhibition of Lipid Accumulation and Oxidation in Hepatocytes by Bioactive Bean Extracts

**DOI:** 10.3390/antiox13050513

**Published:** 2024-04-25

**Authors:** Dya Fita Dibwe, Emi Kitayama, Saki Oba, Nire Takeishi, Hitoshi Chiba, Shu-Ping Hui

**Affiliations:** 1 Faculty of Health Sciences, Hokkaido University, Kita-12, Nishi-5, Kita-Ku, Sapporo 060-0812, Japan; dibwedf@hs.hokudai.ac.jp; 2 Graduate School of Health Sciences, Hokkaido University, Kita-12, Nishi-5, Kita-Ku, Sapporo 060-0812, Japan; emi7-04@hotmail.co.jp (E.K.); saki.oba.h@gmail.com (S.O.); n10461991724nasukakstg@docomo.ne.jp (N.T.); 3 Department of Nutrition, Sapporo University of Health Sciences, Nakanuma Nishi-4-3-1-15, Higashi-Ku, Sapporo 007-0894, Japan; chiba-h@sapporo-hokeniryou-u.ac.jp

**Keywords:** functional foods, bean extracts, polyphenols, hepatosteatosis, neutral lipids, oxidative lipidomics, triacylglycerols, hydroperoxides

## Abstract

During our search for natural resources that can inhibit lipid droplet accumulation (LDA) and potentially prevent metabolic dysfunction-associated fatty liver disease (MAFLD) and its progressive stages, such as metabolic dysfunction-associated steatohepatitis (MASH), eight bean extracts (BE1–BE8) were tested for their ability to inhibit lipid accumulation and oxidation in hepatocytes. Substantial inhibitory effects on LDA with bean extracts (BEs) BE2, BE4, BE5, and BE8 were demonstrated. An advanced lipidomic approach was used to quantify the accumulation and inhibition of intracellular triacylglycerol (TAG) and its oxidized species, TAG hydroperoxide (TGOOH), in hepatocytes under fatty acid-loading conditions. The results show that the antioxidants BE2 and BE8 are potential candidates for regulating TAG and TGOOH accumulation in fatty acid-induced lipid droplets (LDs). This study suggests that bean-based foods inhibit LDs formation by decreasing intracellular lipids and lipid hydroperoxides in the hepatocytes. The metabolic profiling of BEs revealed that BE2 and BE8 contained polyphenolic compounds. These may be potential resources for the development of functional foods and drug discovery targeting MAFLD/MASH.

## 1. Introduction

Chronic diseases are the primary causes of death and disability worldwide. Metabolic dysfunction-associated fatty liver disease (MAFLD) is the most prevalent chronic liver disease [1]. MAFLD is a chronic liver disorder prevalent worldwide, and numerous scientific studies have suggested that oxidative stress can accelerate its development. This occurs through the activation of Kupffer cells, hepatic stellate cells, and hepatocytes, which can trigger the progression of MAFLD to Metabolic dysfunction-associated steatohepatitis (MASH) [2,3]. Therefore, researchers are investigating the potential benefits of antioxidants in MAFLD treatment. Previous studies have shown that reactive oxygen species (ROS) play a role in MAFLD, supporting the use of antioxidants as potential therapies. Oxidative stress is a key factor in the development of MAFLD, its progression to MASH, and the development of hepatic fibrosis, making antioxidants a potential treatment option for MAFLD [4]. The incidence of MASH-related hepatocellular carcinoma (MASH-HCC) and MAFLD-related hepatocellular carcinoma (MAFLD-HCC) has increased significantly worldwide and is becoming the most common cause of liver cancer [5]. At present, there is a lack of effective therapeutic agents for MASH, as well as tools and indicators to prevent and monitor the progression of MASH to HCC. Therefore, it is of great practical importance to strengthen research on the pathogenesis of MASH-HCC and to find effective drugs to prevent and treat NASH-HCC [6,7].

Lipotoxicity plays an important role in MAFLD pathogenesis. Lipotoxicity manifests as excessive accumulation of free fatty acids in the liver. Hepatocytes are subjected to increased uptake of free fatty acids from the bloodstream. Local and circulating free fatty acids are critical causative agents of insulin resistance, hyperlipidemia, inflammation, and hepatic lipidosis [8,9,10,11]. In addition, the major fatty acids present in the human body are palmitic and oleic acids, and these free fatty acids are easily converted to neutral lipid triacylglycerol (TAG) and their oxidized species, namely, triacylglycerol hydroperoxide (TGOOH). Therefore, understanding their roles in intracellular uptake and cytotoxicity is crucial to understanding the mechanisms of MASH [12]. An increasing number of studies have reported an association between excessive intracellular lipid droplet accumulation (LDA) and obesity, diabetes, and other metabolic disorders [13,14]. Hepatic LDA is thought to be involved in the early stages of MAFLD [15]. Inflammation-induced oxidative stress is a common feature of many chronic diseases. Excessive reactive oxygen species cause oxidative damage to important cellular components such as DNA, proteins, and lipid membranes. The hydroxyl radical is highly reactive and rapidly extracts hydrogen atoms from biomolecules in an immediate environment [16]. Phospholipids and membrane triglycerides are the main targets of hydroxyl radical attack and the formation of lipid radicals [17,18]. These lipid radicals are rapidly oxidized, leading to the peroxidation of lipid acyl chains. The excessive intracellular accumulation of neutral lipids and their oxidized species has been associated with various human metabolic diseases such as MAFLD/MASH [19,20]. Agents capable of reducing the accumulation of LDs and oxLDs in hepatocytes/liver via the regulation of neutral lipids TAG and TGOOH represent promising candidates for the prevention and management of MAFLD/MASH and associated diseases. Bioactive compounds of natural origin with LDA inhibitory activity (LDAI) in hepatocytes are potential candidates [12,21,22,23,24,25,26,27].

The inhibition of LDA and lipid oxidation is the main approach for preventing hepatic steatosis. In our search for natural crude extracts with LDAI activity, flazin, an alkaloid β-carboline with a C-3 carboxyl group, a C-1 furfuryl alcohol moiety, and its derivative with a piperidine C-ring from oyster extracts were identified as LDAI molecules. They showed significant LDAI activity through the regulation of LDs accumulation. They significantly inhibited the accumulation of TAG species in cells, activated lipolysis, and suppressed lipogenesis. These results suggest that β-carboline alkaloids may be potentially useful in preventing MAFLD. Crude extracts, including medicinal plants and functional foods, are potential sources of LDAI candidates [12,28]. Therefore, functional foods and nutraceuticals may be beneficial in the management of chronic diseases, including MAFLD/MASH, and offer a potential target for drug discovery and developments. Following this approach, we collected familiar beans from the Hokkaido region for use as edible foodstuffs. We examined their effects on hepatocytes as a source of potential natural bioactive substances regulating LDAI, TAG and TGOOH [12,28,29]. Beans are essential crops used as the main vegetable in many countries. Beans, including soybean plants, are rich in protein, ω-3 fatty acids, carbohydrates, dietary fiber, and micronutrients such as folate, manganese, vitamin K, potassium, and zinc. Polyphenols and alkaloids have also been identified in these foods and exhibit numerous bioactivities [30]. Thus, the LDAI capacity of beans was assessed under two fatty acid loading conditions, oleic acid (OA) and linoleic acid (LA) in hepatocytes, and the main secondary metabolites in the bioactive samples were analyzed.

## 2. Materials and Methods

### 2.1. Materials

The chemicals and equipment used in this study are described in the supporting information as previously reported [12] (Supplemental Information: Materials and methods). The eight bean samples used in this study were collected by DDF, SO and NT from the Sapporo market in April 2020 and deposited at the Health Innovation Center of the university’s health science faculty (Table S1). They were listed as (Code, Scientific name (abbreviation)): BE1, *Glycine max* (*GM1*); BE2, *Glycine max* (*GM2*); BE3, *Vigna angularis* (*VA*); BE4, *Phaseolus vulgaris* (*PV*); BE5, *Phaseolus coccineus* (*PC1*); BE6, *Phaseolus coccineus* (*PC2*); BE7, *Vicia faba* (*VF*); BE8, *Vanilla planiflolia* (*VP*). To identify the bioactive extracts and metabolites derived from food sources, we prepared eight different food samples, including soybeans, black soybeans, adzuki beans, taisho kintoki, purple kidney beans, white kidney beans, lentils, and vanilla beans (BE1–BE8). Each of these food items was purchased from a market or supermarket in Sapporo in the spring of 2020 (Table S1). First, 10 g of each sample was ground using a mortar and pestle, and the crushed material was transferred to a beaker. Next, 100 mL of methanol was added to the mixture for one hour. The mixture was sonicated for 30 min, which was repeated twice to obtain the extract. Methanol was removed by decompression and dried to obtain the methanol extract. The biological activities of the extracts were evaluated using a lipid accumulation inhibition test.

### 2.2. Evaluation of Cell Viability and Lipid Droplet Accumulation Inhibition Assay

Cytotoxicity and lipocytotoxicity assays were performed according to the manufacturer’s protocol using the Cell Counting Kit-8 (CCK-8; Dojindo Molecular Technologies, Rockville, MD, USA) as described previously [12] (Supplemental Information: Materials and Methods, Figure S1). HepG2 cells were purchased from RIKEN BRC cell bank (Ibaraki, Japan).

LDAI activity was determined using an Oil Red O assay with 24-well plates (*n* = 4 per treatment) as previously reported [12]. LD staining was performed with modifications based on a previous report [12] (Supplemental Information: Materials and Methods). HepG2 cells were seeded in 24-well plates at a concentration of 5.0 × 10^4^/well and incubated for 24 h. Subsequently, the cells were treated with OA (0.25 mM) and BEs samples in serial dilution concentration from 500 μg/mL and incubated overnight. On the following day, oil red O staining was performed. Specifically, the medium was removed and washed twice with 200 μL of PBS. Next, 200 μL of 10% formalin was added and allowed to stand for 10 min. After removing the formalin and washing twice with 200 μL of PBS, 200 μL of 60% IPA was added and allowed to stand for 10 min. The mixture was then removed, and Oil Red O Working Solution (prepared by mixing Oil Red O Stock Solution and DDW in a 6:1 ratio and filtering) was added and allowed to stand for 15 min. The upper layer was removed, washed with 200 μL of 60% IPA, and then washed twice with 300 μL of PBS. Next, 200 μL of 100% isopropyl alcohol (IPA) was added and de-stained. The upper layer (150 μL) was transferred to a 96-well plate, and the absorbance (at 560 nm) was measured using an AR-VO-MX plate reader. LDA and LDAI quantification were performed for all BEs samples. The quantification of accumulation and inhibition under OA was carried out by comparing the LDA under OA treatment with the untreated control (−OA) group and the LDAI under OA treatment with BE2 and BE8 when compared with the untreated control (+OA) group. All LDA and LDAI data were normalized at the end of the process, and the quantification was expressed as a percentage (with six replicates).

### 2.3. Analysis of TAG and TGOOH Species

Lipid extraction. HepG2 cells (2.0 × 10^5^/well) were cultured in a dish for 24 h, and after adding fatty acids and extracts, the cells were cultured for another 24 h. On the third day, the media was removed and the cells were washed twice with 300 µL of PBS. Then, 300 µL of trypsin was added and the cells were incubated for 10 min. Then 300 µL of DMEM was added and the cells were collected in 1.5 mL microtubes. After centrifugation at 13,000 rpm for 15 min at 4 °C (High-Speed Micro Centrifuge himac CF 15R, Tokyo, Japan), the supernatant was separated into new 1.5 mL microtubes. The residue was combined with 150 µL MeOH, 400 µL CHCl_3_ and 10 µL internal standard and vortexed (MSV-3500, Biosan, Riga, Latvia) at a speed of 3500 rpm for 10 min. Then centrifugation at 15,000 rpm for 15 min at 4 °C was performed, and the supernatant was carefully separated into new 1.5 mL microtubes. To the residue, 150 µL MeOH and 400 µL CHCl_3_ were added, and extraction was carried out. The resulting extracts were combined, and the solvent was removed under vacuum (CC-105, Tommy Seiko Co., Ltd., Tokyo, Japan) for about 1 h. To the dried lipids, 100 µL MeOH was added and vortexed at 3500 rpm for 1 min, then centrifuged at 15,000 rpm at 4 °C for 10 min, and the supernatant was transferred to LC-MS vials and stored at −80 °C until the measurement date.

LC-MS measurements were performed on a Shimadzu Prominence HPLC system (Shimadzu Corp., Kyoto, Japan). It was coupled to an LTQ Orbitrap mass spectrometer (Thermo-Fisher Scientific Inc., Waltham, MA, USA). Lipids extracted from HepG2 cells were purified using an Atlantis T3 C18 column (2.1 × 150 mm, 3 μm, Waters, Milford, MA, USA) at a flow rate of 200 μL/min. LC gradient elution was performed using a mobile phase consisting of 10 mM ammonium acetate solution, isopropanol, and methanol. The measurements were carried out in positive mode: the volTGe of the MS capillary was 4.04 kV, the flow rate of the sheath gas (nitrogen) was 50 psi, and the auxiliary gas (nitrogen) was 20 psi. The high-resolution MS data were obtained in a scan range of *m*/*z* 150–1200.

The obtained data were subjected to peak alignment, peak area integration, and quantitative calculations using Thermo Fisher Scientific Xcalibur 2.2. Software with a mass tolerance of 5.0 ppm. The exported quantitative data for TAG and TGOOH lipid species were subjected to further calculation using Excel, then exported to GraphPad Prism V7.0/10.1.2 software for further graph processes and statistical analysis (Supplemental Information: Material and Methods). The data are portrayed in heat maps depicting the accumulated and inhibited TAG and TGOOH species in the cells. The fluctuation of the accumulated TAG and TGOOH species under OA and LA treatment with BE2 and BE8 was quantified and normalized at the end of the process. The accumulation and inhibition of TAG and TGOOH species in the cells were expressed as percentages, and six replicates were used. The graph illustrates the mean values of the accumulation of TAG and TGOOH species in the control (+OA and +LA) group compared with the untreated control (−OA and −LA) groups, and the inhibition of accumulated TAG and TGOOH species in the BE2- and BE8-treated group under +OA and +LA conditions compared with the untreated control (+OA and +LA) groups. The results were statistically significant with *p*-values of **** *p* < 0.0001, *** *p* < 0.001, ** *p* < 0.01 and * *p* < 0.05 (Supplemental Information: Materials and Methods).

### 2.4. Metabolites Profiles

NMR profiling of selected bean samples: Methanol extracts were weighed to 0.6 mg each and dissolved in 600 µL of DMSO-d6 to make 10 mg/mL samples for NMR. ^1^H-NMR proton data were obtained using a 400 MHz JNM-ECX400P (JEOL, Tokyo, Japan). The spectra were processed using JOEL v6.3.0 software, and chemical shift values are expressed in ppm.

HPLC profiling of selected bean samples: Methanol extracts were weighed to 10 mg each and dissolved in 1 mL of methanol to be used for HPLC measurement. The HPLC Solvent System, the mobile phase, consisted of solvents A, Mill-Q, and B, HPLC-grade methanol, at a flow rate of 5 mL/min. For the HPLC method, the gradient method was 0–1 min. The gradient method was 0–1 min, 20% B; 1–20 min, 20–100% B; 20–23 min, 100% B; and a post-run time of 2 min including 25 min, 100–20% B; and stop the measurement. Chromatograms were recorded at 200 nm, 275 nm, 343 nm.

LC-MS profiling of selected bean samples: LC-MS measurements were performed on a Shimadzu Prominence HPLC system (Shimadzu Corp., Kyoto, Japan) coupled to an LTQ Orbitrap system. LC-MS measurements were performed using a Shimadzu Prominence HPLC system (Shimadzu Corp., Kyoto, Japan) coupled to an LTQ Orbitrap mass spectrometer (Thermo Fisher Scientific Inc., San Jose, CA, USA) with an electrospray ionization (ESI) source. The conditions of the LC-MS instrument were as follows: samples of methanol extracts of beans were separated using an Atlantis T3 C18 column (2.1 × 150 mm, 3 μm, 155 Waters, Milford, MA, USA) at a flow rate of 200 µL/min. LC gradient elution was performed using a mobile phase consisting of a 10 mM ammonium acetate solution, isopropanol, and methanol. The measurements were carried out in positive mode: the volTGe of the MS capillary was 4.04 kV, the flow rate of the sheath gas (nitrogen) was 50 psi, and the auxiliary gas (nitrogen) was 20 psi. The high-resolution MS data were obtained in a scan range of *m*/*z* 50–500.

## 3. Results and Discussion

### 3.1. Evaluation of Cell Viability under Co-Treatment with Fatty Acids and Bean Extracts

In our previous study, the viability of HepG2 cells treated with oleic acid (OA) was measured 24 h after exposure to OA. Briefly, we induced lipid droplets by supplementing DMEM with 0.1, 0.25, and 0.1 mM oleic acid in HepG2 cells after incubating for 24 h to determine the appropriate concentration for the study. Lipid droplets were significantly increased, by 2.6- and 2.8-fold, for 0.25 and 0.5 mM. Thus, 0.25 mM of oleic acid was chosen to induce lipid droplets in HepG2 cells. Moreover, HepG2 cells were tested for viability after treatment with 0.25 mM of oleic acid at different concentrations. No cytotoxicity was observed after 24 h of treatment (Figure S1, Scheme S1, capture image of real-time lipid droplet accumulation). Therefore, 0.5 mM was determined to be a non-toxic concentration of OA and the optimal concentration for inducing LDs [12]. Thus, in the present study, cell viability was assessed 24 h after treatment with the eight different bean extracts at 125–500 µg/mL concentrations. The results show that the IC_50_ values were above 500 µg/mL for all samples (Figure S1). Based on these results, 500 µg/mL was selected as the final concentration of bean extract for the LDAI assay.

### 3.2. Lipid Droplet Accumulation Inhibition Activity of Selected Bean Extracts

**Figure 1:**
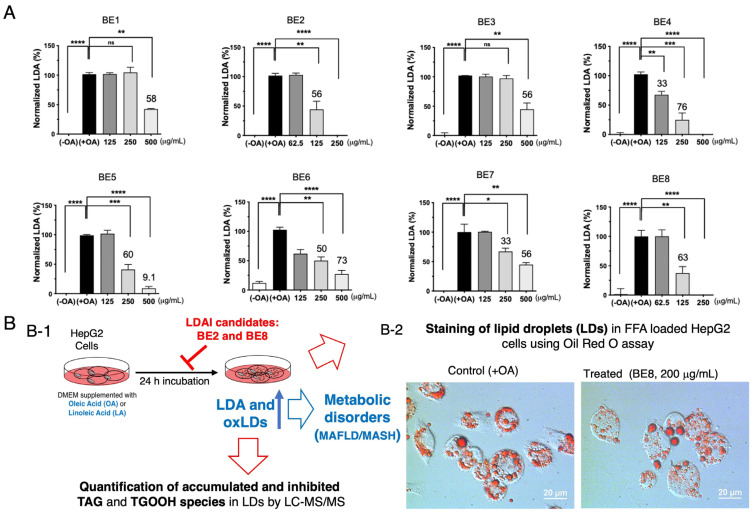
(**A**) LDAI activity of selected bean extracts on OA-loaded HepG2 cells. Graph showing the mean values of the LDAI (four replications). **** *p* < 0.0001, *** *p* < 0.001, ** *p* < 0.01, * *p* < 0.05 and ns not significance, one-way analysis of variance (ANOVA) with Tukey’s multiple comparisons test when compared with the untreated control. (**B**) (**B-1**) Discovery of the LDAI candidate strategy: Quantification of TAG and TGOOH accumulation and inhibition in LDs using Orbitrap-LC-MS/MS analysis under OA and LA conditions. (**B-2**) LDAI activity of bioactive BE8 samples in HepG2 cells using an Oil Red O staining technique: phase contrast images showing LDs (red) in control (OA) and treated BE8 cells. The blue colors indicate free fatty acids (OA and LA) used in this study; the small blue arrow indicates the augmentation of LDA and oxLDs in HepG2 cells, and the big blue arrow indicates the metabolic disorders induced in LDA and oxLD conditions. The red colors indicate LDAI candidates that inhibit LDA and oxLDs, as illustrated by the red inhibition mark; the large red arrows indicate quantification of fluctuation of TAG and TGOOH under OA and LA conditions using LC-MS, and Oil Red O staining of LDs.

**Figure 2:**
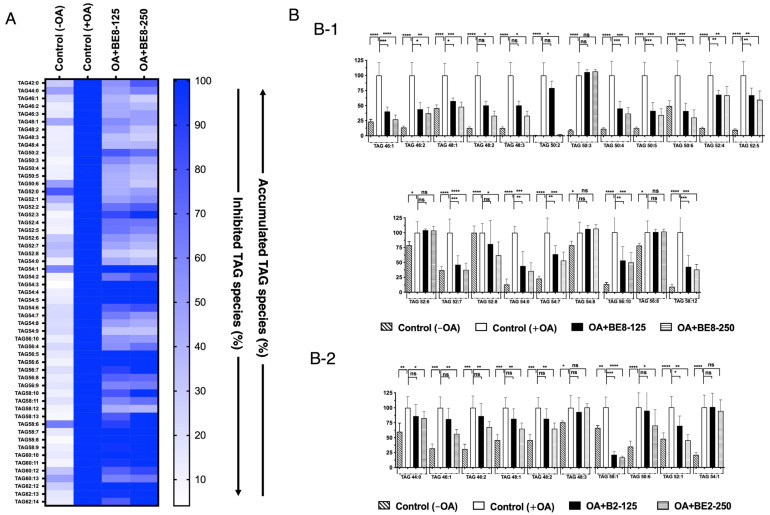
(**A**) Heat map of accumulated and inhibited TAG species in cells. (**B**) Quantification of fluctuation of accumulated TAG species under OA treatment. Analysis of triacylglycerol molecular species under OA treatment with: (**B-1**) BE8 sample and (**B-2**) BE2 sample. Graph showing the mean values of the LDA and LDAI (six replications) **** *p* < 0.0001, *** *p* < 0.001, ** *p* < 0.01, * *p* < 0.05 and ns not significance, one-way analysis of variance (ANOVA) with Tukey’s multiple comparisons test when compared with the untreated control (+OA) group. ns, not significant; TAG, triacylglycerol; OA, oleic acid.

Our previous report revealed that the accumulation of intracellular LDs increases in a concentration-dependent manner under OA conditions. Lipid droplets were significantly induced at a factor of 2.6 and 2.8 for 0.25 and 0.5 mM, respectively. Based on these results, we decided to use 0.25 mM of oleic acid to induce lipid droplet formation in HepG2 cells. HepG2 cells were incubated for 24 h under OA loading (0.1~0.5 mM in the non-toxic range) [12]. In particular, LD accumulation predominantly increased at 0.25 mM and above. The real-time observation of this phenomenon showed that intracellular LD levels increased while the cells were still alive [12]. Based on these results, in the present study, the conditions to induce steatosis in HepG2 cells were set at 0.25 mM OA and 24 h of incubation. Eight selected bean extracts were simultaneously co-treated with 0.25 mM OA and incubated for 24 h. The results are shown in Figure 1A (Scheme S2). All bean samples showed a decreasing trend in LD. In particular, samples BE2, BE4, BE5, and BE8 exhibited a pronounced inhibition of lipid droplet accumulation with LDAI values of 56% at 125 µg/mL and 100% at 250 µg/mL for BE2; 33% at 125 µg/mL and 76% at 250 µg/mL for BE4; 60% at 250 µg/mL and 9.1% at 500 µg/mL for BE5; and 63% at 125 µg/mL and 100% at 500 µg/mL for BE8 (Figure 1). To visualize the inhibition of neutral lipid accumulation in lipid droplets (LDs), we stained LD formation in control +OA and LDAI experiments treated with BE2 and BE8 samples in HepG2 cells loaded with oleic acid. The formation and inhibition of LDs, as well as cell morphological changes, were observed at 24 h by staining with Oil Red O. The significant accumulation of LDs was observed at 24 h after incubation, as demonstrated by imaging with LDA and Oil Red O staining (Figure 1B(B-1,B-2)). LDA was observed in control cells and inhibited in treated samples after 24 h. Both the control and treated groups were used to determine the LDAI activity of BEs samples in HepG2 cells using Oil Red O staining of LDs. In the treated BE8 sample, the number of accumulated LDs was significantly reduced at 24 h, with a clear change in the morphology and size of the LDs compared to those in the control at the corresponding time points, as shown in Figure 1(B-2). The quantification of TAG and TGOOH accumulation in control OA and LA and inhibition by BE2 and BE8 in LDs was performed using Orbitrap-LC-MS/MS analysis under OA and LA conditions.

### 3.3. Quantification of the Effect on Inhibiting the Accumulation of TAG and TGOOH Species

The accumulation of hepatic lipids, primarily in the form of triacylglycerols (TAGs), is a defining characteristic of MAFLD. Researchers have implicated oxidative stress, as evidenced by the presence of lipid oxidation products, in the progression of more severe forms of MAFLD, such as MASH, and associated diseases [31]. To obtain more accurate information on how changes in molecular species occur, researchers have recently reported the limitations of traditional methods that rely solely on total oxidation determination, and the importance of adopting advanced approaches that overcome these limitations. These investigations may lead to a better understanding of the diagnosis, prevention, and treatment of MAFLD, including MASH and associated diseases. In our ongoing studies, we measured and analyzed fluctuations in TAG and TGOOH species in FFA-loaded HepG2 cells induced by food-derived bioactive compounds. Starting with the abundant individual FFA under physiological conditions, researchers used PA-, OA-, and LA-loaded HepG2 cells to compare traditional, unique imaging, and lipidomics approaches. TAG levels for PA, OA, and LA were observed at 6 h A, 12 h B, 24 h C, and 48 h D, while TGOOH levels for PA, OA, and LA were observed at the same time points. This opens up opportunities to investigate fluctuations in food-derived metabolites that target the regulation of pronounced accumulated TAG and TGOOH neutral lipid species in lipid droplets. Among the BEs samples, BE2 and BE8 significantly inhibited excessive lipid droplet accumulation. Thus, both bioactive LDA extracts were subjected to a detailed investigation of their effects on TAG and TGOOH neutral lipid species in the LDs. In this study, the fluctuation in TAG and TGOOH neutral lipid species induced in lipid droplets of OA- and LA-loaded HepG2 cells after treatment with BE2 and BE8 bioactive samples was quantified in detail.

In our previous study, we assessed the antioxidant capacity of eight selected bean extracts (BE1–BE8) using a DPPH radical scavenging assay. Among these extracts, BE3, BE-7, and BE-8 demonstrated the highest activities, with values of 5.96 nmolTE/mg, 7.38 nmolTE/mg, and 7.60 nmolTE/mg, respectively. Additionally, we evaluated the total phenolic content (TPC) of the eight selected bean samples (BE1–BE2). The results reveal that both BE5 and BE8 showed the highest phenolic contents at 52 and 60 μg/mg, respectively. This finding suggests that the observed antioxidant activity of BE8 may be attributed to the high concentration of phenolic compound derivatives. Previous research has also suggested that phenolic metabolites play a role in preventing metabolic diseases such as MAFDL/MASH. In this study, we tested the ability of the bioactive LDAI extracts to inhibit both neutral lipids TAG and oxidized species of TGOOH, which are implicated in metabolic disorders through oxidative stress [12,28,32].

#### 3.3.1. Analysis of Accumulated TAG and TGOOH Species Induced by Oleic Acid in Hepatocytes

OA-treated HepG2 cells showed a significant increase in LDA, and a significant LDAI was observed for the BE2 and BE8 samples, as shown in Figure 1A. Bioactive BE2 and BE8 samples inhibited LDA in a concentration-dependent manner in HepG2 cells treated with oleic acid. We analyzed the effects of LDAI bioactive bean extracts on TAG species accumulation in hepatocytes using LC-MS. In this study, BE8 (0.25 mM and 0.125 mM) significantly inhibited LDA in HepG2 cells loaded with 0.25 mM OA at 100% and 56%, respectively. The profiles of neutral lipid TAG and TGOOH species in cells incubated for 24 h were analyzed using Orbitrap LC-MS. All the accumulated TAG and TGOOH species induced by OA were detected (Supplementary Materials, Tables S2 and S3). Seventy-five triacylglycerol molecular species were detected using an in-house lipidomic system. More than 30 species were accumulated. Eighteen of the accumulated species were inhibited by BE8 treatment (Figure 2(B-1)). BE8 inhibited the accumulation of seventeen and eighteen TAG molecular species in cells after 24 h of treatment at 125 and 250 µg/mL, respectively. Most of the accumulated TAG molecular species were inhibited by BE8 in the range of 25–49%, except for TAGs 52:4, 52:5, 52:8, 54:7, and 56:10. Eight accumulated TAG molecular species (TAGs:44:0, 46:1, 46:2, 48:1, 48:2, 50:6, 52:1, and 54:1) were inhibited by BE2 by 50–74%. The accumulated triacylglycerol molecular species (TAG 50:1) were particularly inhibited by BE2 in the range of 25% to 49%.

Fourteen accumulated TAG molecular species were inhibited by BE8 at concentrations ranging from 25 to 49% (TAGs 46:1, 46:2, 48:1, 48:2, 48:3, 50:2, 50:4, 50:5, 50:6, 58:12, 56:10, 52:7, 54:0, and 58:12). Between 50% and 74%, significant inhibition was observed, especially by BE2, for eight accumulated TAG molecular species (TAGs:44:0, 46:1, 46:2, 48:1, 48:2, 50:6, 52:1, and 54:1) and only for five accumulated TAGs molecular species (TAGs 52:4, 52:5, 52:8, 54:7, and 56:10) using the BE8.

Among all accumulated triacylglycerol molecular species, total inhibition was observed only for TAGs 50:2 during BE8 treatment (Figure 2(B-1)). BE2 and BE8 ameliorated LDA by inhibiting the accumulation of triacylglycerol molecular species in human HepG2 cells (Figure 2(B-1,B-2)).

**Figure 3:**
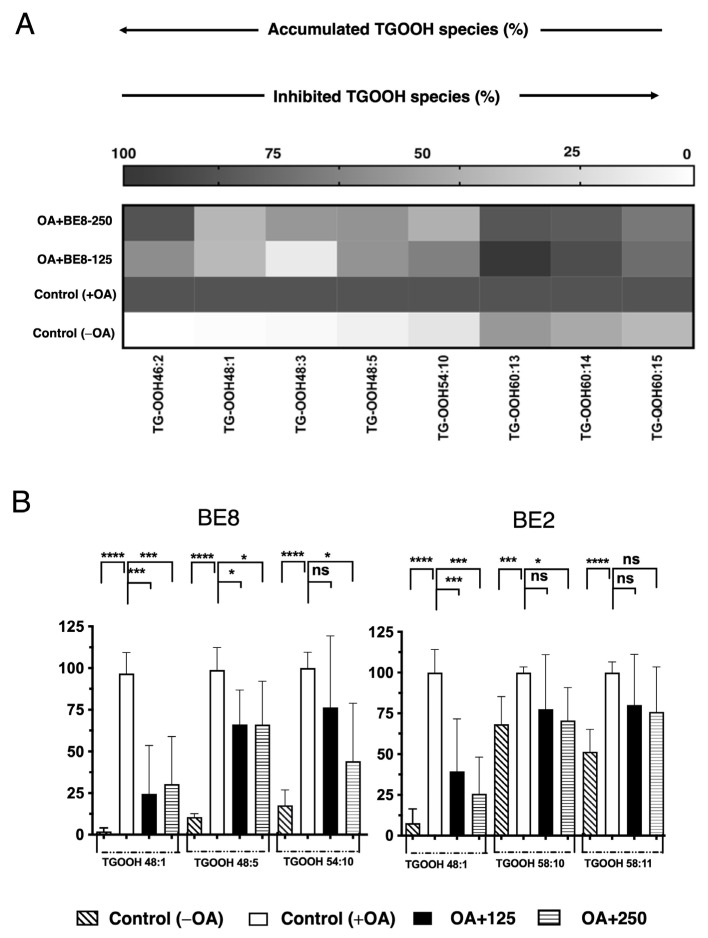
(**A**) Heat map of accumulated and inhibited TGOOH species in cells. (**B**) Quantification of fluctuation of accumulated TGOOH species under OA treatment. Analysis of TAG molecular species under OA treatment with BE2 and BE8. Graph showing the mean values of the LDA and LDAI (six replications) **** *p* < 0.0001, *** *p* < 0.001, * *p* < 0.05 and ns not significance, one-way analysis of variance (ANOVA) with Tukey’s multiple comparisons test, when compared with the untreated control (+OA) group. ns, not significant; TGOOH, triacylglycerol hydroperoxide; OA, oleic acid.

In addition, the fluctuation of the TGOOH species was analyzed under OA conditions. As a result of this analysis, eight TGOOH molecular species accumulated under the OA treatment (Figure 3A and Table S3). Furthermore, among the eight species, three accumulated TGOOH molecular species (TGOOHs 48:1, 48:5 and 54:10) decreased significantly after BE8 sample treatment. The BE2 sample treated under OA conditions showed only a tendency to decrease two TGOOH species (TGOOHs 58:10 and 58:11) (Figure 3B). Among the accumulated TGOOH molecular species TGOOHs 48:1 and 54:10 were significantly inhibited by both BE2 and BE8 under OA conditions.

#### 3.3.2. Quantification of TAG and TGOOH Species Induced by Linoleic Acid

##### From Traditional and Imaging Approaches to Advanced MS Approaches 

A fundamental first step in lipid oxidation is the formation of lipid hydroperoxides, which possess an added hydroperoxyl group and undergo C=C double bond rearrangement. Depending on the source of the oxidation, such as radical oxidation (including thermal oxidation and auto-oxidation), enzymatic oxidation (including lipoxygenase), and singlet oxygen oxidation (including photo-oxidation and inflammation), the generated hydroperoxides (-OOH) exhibit distinct and specific features. These reactions subsequently lead to a series of continuous chain reactions [32,33].

We focused on advanced methods and strategies for oxidative lipidomics to fill the gap in the traditional approaches to oxidized lipid analysis in LDs lipidome. Typically, the analysis of oxidized lipids is based on the indirect investigation of lipid oxidation end products, which can be measured using colorimetric assays, immunoassays, or electron spin resonance. These methods include thiobarbituric acid-reactive substances (TBARS) and malondialdehyde (MDA) tests, ferrous oxidation in xylenol orange (FOX), and gated diene tests. However, the results obtained from these methods, including recent fluorescence approaches, are usually considered an indicator of the degree of lipid oxidation rather than as detailed analyses of changes in lipid species. To address this limitation, the MS/MS-based detection of oxidized molecular lipid species has emerged as a promising approach [32,33,34,35].

##### MS/MS-Based Detection of Oxidized Molecular Lipid Species

MS technology addresses the issues of sensitivity, specificity, accuracy, and dynamic range to a large extent, and enables high-throughput analysis, whereas other methods may require selection and adaptation to measure lipid oxidation products. This is particularly beneficial for the detection of oxidized lipids, generated in complex mixtures with varying levels and structural diversities. The MS-based detection of oxidized lipids is emerging as a promising approach [33].

**Figure 4:**
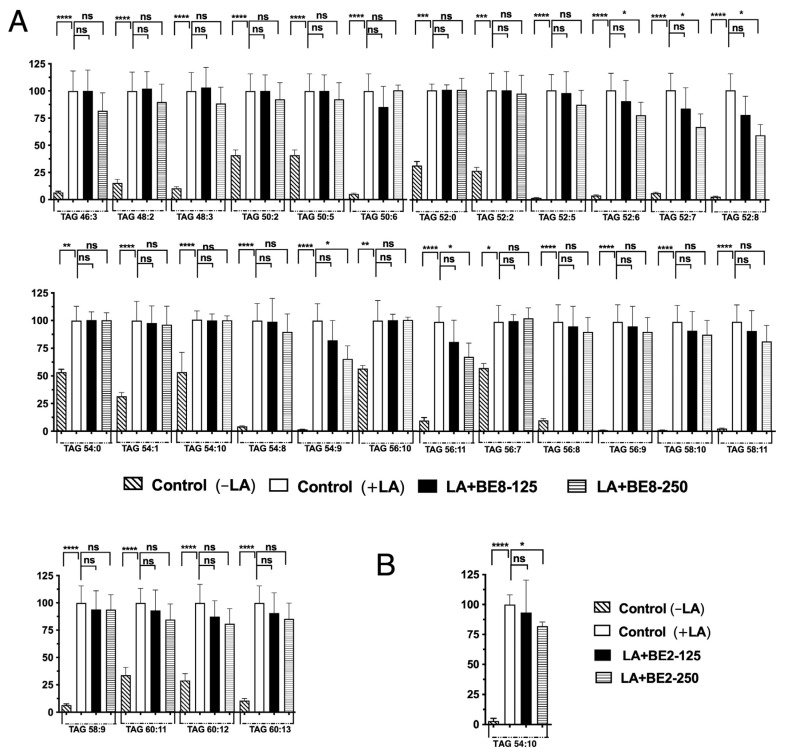
Quantification of the fluctuation of accumulated TAG species under LA treatment. Analysis of triacylglycerol molecular species under OA treatment with (**A**) BE8 and (**B**) BE2. Graph showing the mean values of the LDA and LDAI (six replications) **** *p* < 0.0001, *** *p* < 0.001, ** *p* < 0.01, * *p* < 0.05 and ns not significance, one-way analysis of variance (ANOVA) with Tukey’s multiple comparisons test. when compared with the untreated control (+LA) group. ns, not significant; TAG, triacylglycerol; LA, linoleic acid.

Currently, LC-MS techniques, particularly LC coupled with tandem MS (LC-MS/MS), are the dominant approaches for oxidative lipidomic applications. Oxidized lipids are structurally characterized by the presence of extra oxygen atoms attached to lipid molecules, such as the insertion of peroxyl/hydroperoxyl/epoxyl groups or deletion of C=C double bonds. By using MS, particularly high-resolution MS, lipid molecules with oxidized functional groups can be captured. MS/MS can then be used for structural confirmation, including the confirmation of the presence of oxygen, the determination of the oxidizing position, and the discovery of specific fragmentation from precursor ions to product ions [33]. Our previous report indicated that linoleic acid (LA) induced LDA and oxLDs in hepatocytes [32]. A unique imaging approach under FA-loaded conditions was reported in relation to conventional approaches for determining the degree of oxidation, where a fluorescence microscopic method for characterizing the size, quantity, and oxidation of lipid droplets (LDs) in HepG2 cells was developed. LDs were induced by palmitic acid (PA), oleic acid (OA), or linoleic acid (LA), and stained with two fluorescent probes for neutral lipids and lipid peroxide; each fatty acid increased the number of LDs and oxidized LDs (oxLDs) and the degree of LD oxidation in a time-dependent manner, as well as increasing intracellular triglyceride hydroperoxide levels. LDs induced by LA without 2,2′-azobis(2-amidinopropane)dihydrochloride (AAPH) showed the most significant degree of oxidation over PA and OA, especially in large LDs (area ≥ 3 μm^2^, oxLD/LD = 52.3 ± 21.7%). Under these conditions, two food-derived antioxidants (chlorogenic acid and DHMBA) were evaluated, and both significantly improved LD characteristics. Moreover, chlorogenic acid reduced the quantity of large LDs by 74.0–87.6% in a dose-dependent manner, providing a new approach for evaluating the effects of dietary antioxidants on LD characteristics. This novel fluorescence-based method was developed to characterize the number, size distribution, and degree of oxidation of LDs in human hepatocytes. Two fluorescent probes were used to individually analyze intact and oxLDs. TG hydroperoxides (TG-OOH) were determined in hepatocytes using liquid chromatography–mass spectrometry (LC–MS)/MS for comparison with the proposed method, and to determine the changes in molecular species to complete the information on the degree of oxidation. The above information shows the differences between the effects of two antioxidants, chlorogenic acid and DHMBA, on the changes in intracellular lipid species of LDs [32]. Thus, following this new finding, an experiment similar to that on the OA-loaded HepG2 condition was performed by replacing fatty acids with LA to analyze in detail the changes in the fluctuation of accumulated TAG and TGOOH induced by the BE2 and BE8-LDAI extracts. All accumulated TAG and TGOOH species induced by LA were detected by Orbitrap LC-MS (Supplementary Materials, Tables S4 and S5). Fifty-seven TAG species were significantly increased in the positive control (+LA) compared with the negative control (−LA) (Figure 4). During treatment with BE8 under LA conditions, TAGs of the five molecular species decreased in a concentration-dependent manner. The most commonly reduced species was TAG 54:9. During treatment with BE2 under LA conditions, TAG 54:10 levels decreased in a concentration-dependent manner, as shown in Figure 4.

**Figure 5:**
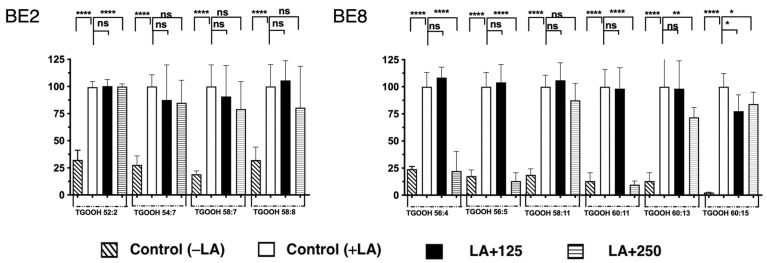
Quantification of fluctuation of accumulated TGOOH species under LA treatment. Analysis of triacylglycerol molecular species under LA treatment with BE2 and BE8. Graph showing the mean values of the LDA and LDAI (six replications) **** *p* < 0.0001, ** *p* < 0.01, * *p* < 0.05 and ns not significance, one-way analysis of variance (ANOVA) with Tukey’s multiple comparisons test when compared with the untreated control (+LA) group. ns: not significant; TGOOH, triacylglycerol hydroperoxide; LA, linoleic acid.

In addition, the fluctuation of the TGOOH species was analyzed under LA conditions. The results reveal that approximately fifteen molecular TGOOH species were detected under LA conditions (Supplementary Materials, Figure S4). In addition, among the fifteen species, three accumulated molecular species (TGOOHs 56:4, 56:5, and 60:11) were reduced during the treatment with BE8. The greatest reduction was observed for the TGOOH60:11. However, during treatment with BE2, only the TGOOH52:2 level was significantly reduced. Compared to the results of BE2 and BE8, no common TGOOH species were reduced in either the BE2 or BE8 samples (Figure 5). Under both OA and LA conditions, the BE2 experiment demonstrated inhibition ranging from 0–49% of one TAG in OA and no inhibition in LA, and in the range of 50–85%, inhibition of eight TAGs was observed in OA, but only one TAG in LA. The BE8 experiment showed inhibition results ranging from 0–49% of 14 TAGs in OA and 1 TAG in LA, and in the range of 50–85%, inhibition of 4 TAGs in OA and 14 TAGs in LA, as shown in Table 1. The evaluation of TGOOH species’ inhibition by BE2 under OA and LA conditions showed a range of 0–49% inhibition for one in OA and no inhibition of TGOOH in LA, and a range of 50–85% inhibition for one TGOOH species under OA and no significant inhibition in LA. Meanwhile, BE8 under the same conditions showed a range of 0–49% inhibition for two TGOOH species in OA and three in LA, and a range of 50–85% inhibition for one TGOOH under OA and three in LA as shown in Table 2. The TGOOH comparison results for BE2 and BE8 under OA and LA conditions showed that both BE2 and BE8 significantly reduced TGOOH 48:1 and 58:10 under OA conditions. However, in LA, BE8 was more effective in reducing TGOOH, whereas no significant inhibition was observed for BE2. Therefore, it was suggested that the BE8 may reduce lipid droplets by inhibiting lipid accumulation and their oxidized species in hepatocytes. BE2 was studied in the same manner to compare the components, and it was found that both BE2 and BE8 reduced several different neutral lipids and their oxidized species. These results suggest that different secondary metabolites may be involved in the regulation of LDA by BE2 and BE8 samples.

The MS/MS-based detection of oxidized molecular lipid species enabled us to clearly observe fluctuations induced by BE2 and BE8 LDAI-bioactive food extracts in individual minor and major accumulated lipid molecular species of TAG and TGOOH. This method allowed for sensitive and accurate quantification with clear discrimination between the two OA- and LA-loading conditions. By extending these results to various FFAs and their combinations, we may gain more insights into the mechanism and diagnosis of oxidative stress via LOOH molecular species. This could potentially expand our understanding of the prevention and discovery of MAFLD/MASH and related metabolic disorders.

### 3.4. Metabolite Profiling of Bioactive Extracts of Beans

Research on the bioactive compounds present in medicinal plants and edible resources, such as crude natural and food extracts, may lead to the discovery of known or new molecules with potential biological activity. Therefore, this method may be beneficial for drug discovery and development. To achieve this, researchers are utilizing metabolomics to identify and profile the chemical components of these resources with the aim of identifying lead molecules for future drug development, as well as the development of functional foods and nutraceuticals. We screened 50 varieties of vegetables, alkaloids, and phenolic compounds for LDAI activity in FFA-loaded cells. Of these, four BEs samples showed significant LDA activity. Thus, this strategy has been applied to bioactive bean extracts to prevent excessive LD formation in cells, which may serve as a lead molecule for drug discovery and development. The significance of combining bioactive metabolites and their key metabolic signatures in functional dietary foods and supplements using NMR and LC-MS metabolomics and complementary techniques, such as diagnostic fragmentation, has been emphasized. BEs samples were compared using NMR and LC-MS. Furthermore, the results of this approach allow for the targeted isolation of key metabolites, and provide a range of possibilities for synthetic routes for LDAI drug discovery and development, as there are currently no chemotherapeutic compounds available.

^1^H-NMR analyses of the BE1–BE8 bean samples indicated the presence of polyphenols as glycosides and aglycone metabolites as one of the chemical constituent classes. Additionally, HPLC analysis of the selected samples revealed prominent peaks in BE8 that were not present in the other samples, including BE2, which was consistent with the NMR and LC-MS results (Supplementary Materials, Figures S2–S11). Previous research has suggested that vanilla beans (*V. planifolia*) contain various components, including phenolic acid derivatives [30]. In this study, four LDAI-bioactive bean extracts were analyzed using LC-MS for metabolite profiling. Chemical metabolite screening from the LC-MS/MS datasets of BE2, BE4, BE5, and BE8 was previously investigated and indicated unknown compounds in molecular networking. In this study, a complementary analysis using diagnostic fragmentation filtering was performed. The results for all four bioactive extracts are presented as 3D images (Figure S9). Several minor phenolic compounds and polyphenolic metabolites (**1**–**11**) were identified from the LC-MS data of the bioactive BEs (Table 3, Figures S9). The results show that seven phenolic compounds were detected in BE8. These compounds were classified into acid and aldehyde groups. Vanillin, one of the major components of the BE8 sample, was an aldehyde, and its presence was indicated by both the LC-MS/MS and NMR data (Figures S5 and S11). In contrast, four polyphenolic metabolites (**7**–**11**) were identified in the BE8 as its minor constituents. Total polyphenol measurements showed that the BE8 sample contained 60 µg of polyphenols in 1 mg. Five polyphenolic metabolites were detected in BE2, four in BE4, and three in BE5. The chemical metabolite analysis of extracts BE2 and BE8 revealed that both extracts are rich sources of minor polyphenolic compounds that could be promising targets for the discovery of candidate LDA and oxLD inhibitors. Our previous report revealed that both BE5 and BE8 samples have the highest phenolic contents, with total phenolic content (TPC) values of 52 and 60 mg/mg, respectively, among the eight selected bean samples (BE1–BE8). This finding suggests that the observed antioxidant activity in BE8 may be attributed to the high concentration of phenolic compound derivatives. Previous research has also suggested that phenolic metabolites play a role in preventing metabolic diseases such as MAFDL/MASH. These results suggest that bioactive bean extracts could be a potential resource for the development of nutraceuticals and furthering natural drug discovery for the prevention and management of MAFLD/MASH and oxidative stress-related diseases [28].

### 3.5. Strengths, Limitations and Future Prospects of the Oxidative Lipidomic Approach Based on the LDAI Study

Additional research is required to assess the bioavailability of bioactive metabolites from various natural and food extracts. Examining the relationship between the structures of these natural compounds and their biological activities may offer valuable insights into their potential for use as effective interventions for preventing and treating the accumulation of LDs and oxLDs via TAG-neutral lipid accumulation and TGOOH oxidized species in hepatocytes.

Advanced lipidomics approaches are powerful and more appropriate for the detection of oxidized intracellular neutral lipid species of lipid droplets, mainly TAGs and their hydroperoxide species, because they can accurately characterize the neutral lipids of individual species and their lipid hydroperoxide (LOOHs) species using mass fragmentation MS2; in contrast, traditional approaches mainly indicate the degree of oxidation instead of the changes occurring in molecular lipid species. Molecular species of LOOHs are prominent non-radical intermediates of lipid peroxidation in both model systems and cells. Advanced lipidomic approaches can fill the gap in information regarding the value of total lipid accumulation and oxidation, and provide valuable potential diagnostic biomarker candidates for more detailed mechanistic investigations. Recently, cholesterol-derived hydroperoxides (ChOOHs) and intracellular neutral lipids such as TGOOHs and CEOOHs have been targeted in this regard for both model systems and cells to clarify their potential effects on prevention, or their use in drug discovery and development investigations connected to metabolic deregulation, by also revealing the capacity of this approach to address the limitation of the degree of oxidation achievable by conventional methods, and providing more sensitive and accurate comprehensive changes as well as the quantification of molecular lipid species under oxidative stress, as detailed in the following four selected reports among our ongoing studies on oxidative lipidomics [32,33,34,35].

Oxidative stress and other disorders, such as cardiovascular diseases, neurodegenerative diseases, and cancers, can be detected using oxidized lipids as potential markers. Lipidomic analysis, which is an advanced approach for studying lipid molecules, is beneficial for biomarker discovery, clinical diagnosis, and pathway exploration. Recent advances in mass spectrometry technology have made it possible to extend the investigation of oxidized lipids, known as oxidative lipidomics. This technique combines the complexity of lipidomics with a focus on oxidation, making it more challenging than regular lipidomics, particularly for sample preparation, standards, and spectral analysis. Despite these challenges, oxidative lipidomics has been applied to clinical and animal experiments to study various diseases. Hui et al. recently described the methods and strategies used in oxidative lipidomics and summarized their current applications [33]. Thus, the development of chromatographic–MS spectrometric technology is expected to further our understanding of the relationships among lipids, oxidation, and human health. A recent study by our research group examined the lipidomic profile of a MASH mouse model and demonstrated that oxidative stress significantly influences lipid regulation and peroxidation at the molecular level. Lipid hydroperoxides, which are involved in the abnormal cycle of lipid metabolism and the disruption of energy production, have emerged as potential diagnostic markers and evaluation indices of the effects of antioxidants. As crucial metabolic organs, the liver and kidneys exhibit oxidative stress-associated dysregulation of the lipidome, and display organ-specific characteristics. Our findings contribute to a better understanding of the role of oxidative stress in NASH development, by highlighting the effects of oxidative damage-induced lipid hydroperoxidation. Chronic kidney disease (CKD), characterized by the progressive loss of kidney function, is associated with renal lipid droplet (LD) accumulation and oxidative damage. LD quality may contribute to CKD development. This study aimed to examine the chemical makeup of LDs formed in human kidney cells exposed to free fatty acids as sources of LDs and oxidized lipoproteins under oxidative stress. LDs were extracted directly from cells using nanotips, followed by in-tip microextraction, and the LD lipidomic profile was determined using nanoelectrospray mass spectrometry. As a result, free fatty acids elevated the LD lipid content and altered their composition considerably. Oxidized lipoproteins distort the proportion of intact lipids, such as triacylglycerols (TAG), phosphatidylcholines (PC), phosphatidylethanolamines (PE), and cholesteryl esters (CE). Notably, the levels of oxidized lipids, including TAG, PC, and PE hydroperoxides, were significantly increased in a dose-dependent manner. Furthermore, the dysregulation of intact lipids is accompanied by the accumulation of lipid hydroperoxides. This study revealed that lipid oxidation and lipid metabolism dysregulation coexist in LDs in kidney cells, presenting a potential new target facilitating diagnosis and offering fresh insights into CKD [34,35].

The fluorescence imaging approach proposed by Tsuikui et al. has the potential to provide precise physicochemical details of LDs in hepatocytes, which could be beneficial to exploring antioxidants in foods and drugs to prevent and alleviate health issues associated with LD accumulation and lipid oxidation. In that study, chlorogenic acid was found to reduce the numbers of large LDs and oxLDs, possibly through TG hydrolysis rather than antioxidative effects. Therefore, the proposed method could be a valuable tool enabling future research on interactions between antioxidants and LDs. In conclusion, the proposed imaging method has the potential to offer detailed physicochemical information regarding LDs in hepatocytes, which may be useful for exploring antioxidants in foods and drugs to prevent and alleviate health issues involving LD accumulation and lipid oxidation. However, one major limitation of the current study is the technical aspect, and it should be noted that LD metabolism involves several cross-talks between ROS, ER stress, mitochondrial function, and lipogenic and lipolytic enzymes, which may also be involved in LD oxidation. Understanding these interactions may help us gain a better understanding of LDs. Additionally, it is important to note that data from primary cell cultures may differ from those retrieved from cultured cancer cell lines, which may not perfectly represent the physiological conditions. Further studies are required to verify these differences. However, it is noteworthy that HepG2 cells are more suitable for use in the stable screening of antioxidants than primary cells because of the availability and easy growth of HepG2 cells. Thus, the proposed method using HepG2 cells is a promising tool that can be used worldwide [12,28,32]. Finally, an oxidative lipidomics study of food extracts and derived food compounds under individual FFAs and the combination of FFAs to clarify the effect of LDAI on prevention and drug discovery related to MAFLD/MASH and related metabolic disorders was linked to the excessive production of LDs and oxLDs. 

## 4. Conclusions

In this study, the inhibitory effect of bean extract on lipid droplet accumulation in FFA-loaded HepG2 cells was investigated. The results show that BE2, BE4, BE5, and BE8 significantly inhibited lipid droplet accumulation in hepatocytes. In addition, the inhibition of accumulation of TAG neutral lipids and their oxidized species in hepatocytes by most bioactive bean extracts, BE2 and BE8, was quantified using a lipidomic approach. BE2 and BE8 significantly inhibited TAG and TGOOH accumulation under OA and LA conditions. Analysis of the chemical constituents of BE2 and BE8 revealed that both extracts were rich sources of polyphenolic metabolites that could be promising targets for the discovery of LDA and oxLD regulator candidates. Beans may be effective foods owing to the limitation of excessive accumulation of lipid droplets via their intracellular neutral lipids, which may potentially impact the prevention of early MAFLD symptoms and serve as potential sources for drug discovery and development.

## Figures and Tables

**Table 1:** Comparison of inhibited TAG species by BE2 and BE8 under both OA and LA conditions in the range of inhibition.

	Inhibited TAG Species			
Range of Inhibition (%)	OA		LA	
	BE2	BE8	BE2	BE8
0–49	1	14	0	1
50–85	8	4	1	14

**Table 2:** Comparison of inhibited TGOOH species by BE2 and BE8 under both OA and LA conditions in the range of inhibition.

	Inhibited TGOOH Species			
Range of Inhibition (%)	OA		LA	
	BE2	BE8	BE2	BE8
0–49	1	2	0	3
50–85	1	1	0	2

**Table 3:** Detection of phenolic and polyphenolic compounds from bioactive BEs.

Compounds	RT	Ion	Calc. *m*/*z*	Exptl. *m*/*z*	ppm
**1**. Gallic acid hydrate	1.83	[M + H]^+^	171.0288	171.0289	0.58
**2**. Syringic acid	2.16	[M + H]^+^	198.0528	198.0524	−2.02
**3**. 4-Hydroxybenzoic acid	2.1	[M + H]^+^	139.0390	139.0382	−5.75
**4**. Vanillic acid	2.02	[M + H]^+^	169.0495	169.0491	−2.37
**5**. 3,4-Dihydroxybenzoic aldehyde	2.1	[M + H]^+^	139.0390	139.0382	−5.75
**6**. 4-Hydroxybenzoic aldehyde	3.13	[M + H]^+^	123.0441	123.0439	−1.63
**7**. Vanillin	3.13	[M + H]^+^	153.0546	153.0545	−0.65
**8**. Daidzein	2.75	[M + H]^+^	255.0642	255.0650	3.14
**9**. Genistein	2.96	[M + H]^+^	271.0601	271.0599	−0.74
**10**. Anthocyanin derivative	2.24	[M + H]^+^	291.0863	291.0851	−4.12
**11**. Genistein glucoside	2.69	[M + H]^+^	431.0984	431.0991	1.62

## Data Availability

Data are contained within the article and Supplementary Materials.

## Supplementary Materials

The following supporting information can be downloaded at https://www.mdpi.com/article/10.3390/antiox13050513/s1. Supplementary Materials and Methods: 1. Chemicals and instruments; 2. Liquid chromatography/mass spectrometry profiling of vanilla bean extract; 3. Lipid droplet accumulation inhibition assay; 4. Metabolite identification using DFF and ^1^H-NMR analyses of BEs; Table S1: List of selected beans used in this study; Figure S1: Cytotoxicity of selected bean samples in HepG2 cell lines; Table S2: Accumulation of TAG species induced by OA; Table S3: Accumulation of TGOOH species induced by OA; Table S4: Accumulation of TAG species induced by LA; Table S5: Accumulation of TGOOH species induced by LA; Figure S2: ^1^H NMR spectrum of BE2 in DMSO-d6; Figure S3: ^1^H NMR spectrum of BE4 in DMSO-d6; Figure S4: ^1^H NMR spectrum of BE5 in DMSO-d6; Figure S5: ^1^H NMR spectrum of BE8 in DMSO-d6; Figure S6: ^1^H NMR spectra of bioactive extract BEs (BE2 and BE8) in DMSO-d6; Figure S7: ^1^H NMR spectra of bioactive extract BEs (BE2, BE4, BE5 and BE8) in DMSO-d6; Figure S8: A schema of the general data processing workflow of LC-MS data; Figure S9: LC-MS profiling of bioactive bean extracts. (A) Diagnostic fragmentation filtering (DFF) plot for metabolites analysis. (B) 3D visualization of MS data; Figure S10: Identification of vanillin in BE8. (A) Three-dimensional (3D) liquid chromatography/mass spectrometry (LC-MS) of BE8 and vanillin. (B) Plot of the vanillin in BE8 using diagnostic fragmentation filtering (DFF); Figure S11. HPLC profile of selected bean samples BE2 and BE8 at 200 nm. Scheme S1: (A) Capture of the LDA real-time images on HepG2 cells with the interval of 6 h. (B) Vial ability staining with AO and EB. (C) Evaluation of LDA and oxLDs inhibition strategy’ Scheme S2: (A) Comparison of the capture of the LDA under −OA and +OA in real-time images on HepG2 cells with the interval of 12 h. (B) LDAI activity of BE1-BE8. (C) Comparison of the vial ability staining with AO and EB under −OA and +OA.

## Abbreviations

LDA, lipid droplet accumulation; LDAI, lipid droplet accumulation inhibition; MAFLD, metabolic dysfunction-associated fatty liver disease; MASH, metabolic dysfunction-associated steatohepatitis; HCC, hepatocellular carcinoma; oxLD, oxidized lipid droplet; OA, oleic acid; LA, linoleic acid; EB, ethidium bromide; AO, acridine orange; LD, lipid droplet; TAGs, triacylglycerol; TGOOH, triacylglycerol hydroperoxide; FFA, free fatty acid; LC/MS, liquid chromatography/mass spectrometry; NMR, nuclear magnetic resonance; HPLC, high-performance liquid chromatography.
