# Supplementary material for: Inhibition of Lipid Accumulation and Oxidation in Hepatocytes by Bioactive Bean Extracts
Source: Antioxidants (Basel). 2024 Apr 25;13(5):513. doi: 10.3390/antiox13050513 (PMC11118026; doi:10.3390/antiox13050513)
Supplement: Supplementary file 1 [file antioxidants-13-00513-s001.zip › antioxidants-2928549-supplementary.pdf]

Inhibition of Lipid Accumulation and Oxidation in Hepatocytes by Bioactive Bean Extracts

Dya Fita Dibwe, [1] Emi Kitayama,[2] Saki Oba,[2] Nire Takeishi,[2] Hitoshi Chiba,[3] Shu-Ping Hui [*,1]

[1] Faculty of Health Sciences, Hokkaido University, Kita-12, Nishi-5, Kita-Ku, Sapporo 060-0812, Japan;
[2] Graduate School of Health Sciences, Hokkaido University, Kita-12, Nishi-5, Kita-Ku, Sapporo 060-0812, Japan;
[3] Department of Nutrition, Sapporo University of Health Sciences, Nakanuma Nishi-4-3-1-15, Higashi-Ku, Sapporo 007-0894, Japan;
* Correspondences: keino@hs.hokudai.ac.jp; Tel./Fax: +81-11-706-3693

Supporting Information

Table of contents:

Materials and Methods P4

1. Chemicals and instruments P4
2. Liquid chromatography/mass spectrometry profiling of vanilla bean extract P5
3. Lipid droplet accumulation inhibition assay P5
4. *Metabolite identification via DFF and, ^{1}H-NMR analyses of selected bean extracts* P6

Table S1: List of selected beans used in this study P7

Figure S1. Cytotoxicity of selected bean samples in HepG2 cell lines P7

Table S2. Accumulation of TAG species induced by OA P8

Table S3. Accumulation of TGOOH species induced by OA P10

Table S4. Accumulation of TAG species induced by LA P11

Table S5. Accumulation of TGOOH species induced by LA P14

Figure S2. ^{1}H NMR spectrum of BE2 in DMSO-d6 P15

Figure S3. ^{1}H NMR spectrum of BE4 in DMSO-d6 P16

Figure S4. ^{1}H NMR spectrum of BE5 in DMSO-d6 P17

Figure S5. ^{1}H NMR spectrum of BE8 in DMSO-d6 P18

Figure S6. ^{1}H NMR spectra of bioactive extract BEs (BE2 and BE8) in DMSO-d6 P19

Figure S7. ^{1}H NMR spectra of bioactive extract BEs (BE2, BE4, BE5 and BE8) in DMSO-d6 P20

Figure S8. A schema of the general data processing workflow of LC-MS data P21

Figure S9. LC-MS profiling of bioactive bean extracts. (A) Diagnostic Fragmentation Filtering (DFF) plot for metabolites analysis. (B) 3D visualization of MS data P22

Figure S10. Identification of vanillin in BE8. (A) Three-dimensional (3D) liquid chromatography/mass spectrometry (LC-MS) of BE8 and vanillin. (B) Plot of the vanillin in BE8 using diagnostic fragmentation filtering (DFF). P23

Figure S11: HPLC profile of selected bean samples BE2 and BE8 at 200nm P24

Materials and Methods

1. *Chemicals and instruments*

General Experimental Procedures: Nuclear magnetic resonance (NMR) spectra were recorded using a JEOL ECX400 Delta spectrometer with TMS as an internal standard; chemical shifts were expressed as δ values. An LTQ Orbitrap XL mass spectrometer (Thermo Fisher Scientific Inc., San Jose, CA, USA) was used for high-resolution electrospray ionization mass spectrometry (HR-ESI-MS) measurements. Methanol was purchased from Wako. High-glucose Dulbecco's Modified Eagle Medium (DMEM), Dulbecco's phosphate-buffered saline (DPBS), trypsin EDTA, fetal bovine serum (FBS), and penicillin-streptomycin (100 U/mL) were purchased from Gibco (Life Technologies, Carlsbad, CA, USA). Other materials used for cell culture were purchased from Corning (NY, USA). NMR spectra were acquired using a 400 MHz JNM-ECX400P spectrometer (JOEL, Japan). The spectra were processed using JOEL software, and the chemical shift (δ) values were expressed in ppm. OA was purchased from Cayman Chemical (Ann Arbor, MI, USA), and absorbance was measured using ARVO-MX (Perkin Elmer, Waltham, MA, USA).

2. *Liquid chromatography/mass spectrometry profiling of vanilla bean extract*

LC-MS instrument conditions

Samples of methanol BEs were separated using an Atlantis T3 C18 column (2.1×150 mm, 3 μm, 155 Waters, Milford, MA, USA) at a flow rate of 200 μL/min. LC gradient elution was performed using a mobile phase of 10 mM ammonium acetate solution, isopropanol, and methanol. The measurements were carried out in positive mode; the voltage of the MS capillary was 4.04 kV, flow rate of the sheath gas (nitrogen) was 50 psi, and auxiliary gas (nitrogen) was 20 psi. The high-resolution MS data were obtained in a scan range of m/z = 150–1100. MS/MS spectra were obtained by data-dependent acquisition using collision-induced dissociation (CID) in ion-trap mode for low-resolution masses. The raw data were processed using Xcalibur 2.2 (Thermo Fisher Scientific Inc., San Jose, CA, USA).

3. *Lipid droplet accumulation inhibition assay*

LDAI activity was determined using an Oil Red O assay with 24-well plates (n = 4 per treatment) based on a First, the staining of LDs in cultured hepatocytes was performed according to the manufacturer's instructions. Next, HepG2 cells (1.5×10^4/well) were supplemented with 10% FBS, cultured, seeded into 35 mm dishes, and treated with the tested samples after 24 h. Oil Red O, a fat-soluble dye, is widely used for staining neutral lipids in LDs. Next, quantification of LD inhibition was assessed for test BEs by comparing it to the untreated control group (+OA) and normalizing the LDA absorbance values (%). Staining was performed using LD staining assay.

4. *Metabolite identification via DFF and, ^{1}H-NMR analyses of selected bean extracts*

Natural products are usually synthesized as mixtures of structurally similar compounds instead of as a single compound. Because of their shared structural characteristics, numerous compounds of the same class are subject to similar MS/MS fragmentation and exhibit several identical product ions and/or neutral losses. The objective of DFF is to accurately detect all compounds of a specific class in a complex extract by filtering out non-targeted LC-MS/MS datasets for MS/MS spectra that contain class-specific product ions and/or neutral losses. The LC/MS profiling of the methanolic extract was presented as a 3D LC/MS plot with retention time and MS values. The LC/MS measurement and analysis of the vanilla BE revealed phenolic acid derivative metabolites.

It was possible to identify the phenolic derivative metabolites as major chemical constituents of the extract compared to the in-house standards. DFF is a straightforward and rapid strategy for detecting entire classes of compounds in a mixture of natural food products. DFF is especially relevant for the dereplication and discovery of natural product compounds. DFF was used for screening metabolites from LC-MS/MS datasets of BE8. The complementary results are 3D images and their DFF. The identified vanillin was further detected in the 3D LC-MS comparison of BE8 and vanillin standard and by using the DFF approach, as shown in the graph with the characteristic ion products and precursors from the LC-MS/MS analysis. As shown in the 3D plot in Figure S10, vanillin was found in the extract based on the retention time and MS/MS spectra of the authentic standard.

Statistical Analysis. All statistical analyses were performed using GraphPad Prism V7.0/10.1.2 software, Multiple comparison tests were performed using one-way ANOVA with Tukey’s multiple comparison test. The level of significance was set at 5%. All values are expressed as the mean ± standard deviation (SD).

Table S1. List of selected beans used in this study

No.	Japanese name	Scientific name	Family	Collection	abbr.	Code
1	Daizu	*Glycine max*	Fabaceae	Sapporo, Hokkaido,	GM	BE1
2	Kuro daizu	*Glycine max*	Fabaceae	Sapporo, Hokkaido	GM	BE2
3	Azuki	*Vigna angularis*	Fabaceae	Sapporo, Hokkaido	VA	BE3
4	Taisyo kintoki	*Phaseolus vulgaris*	Fabaceae	Sapporo, Hokkaido	PV	BE4
5	Murasaki hanamame	*Phaseolus coccineus*	Fabaceae	Sapporo, Hokkaido,	PC	BE5
6	Shiro hanamame	*Phaseolus coccineus*	Fabaceae	Sapporo, Hokkaido,	PC	BE6
7	Soramame	*Vicia faba*	Fabaceae	Sapporo, Hokkaido,	VF	BE7
8	Vanilla bean	*Vanilla planiflolia*	Orchidaceae	Sapporo, Hokkaido,	VP	BE8

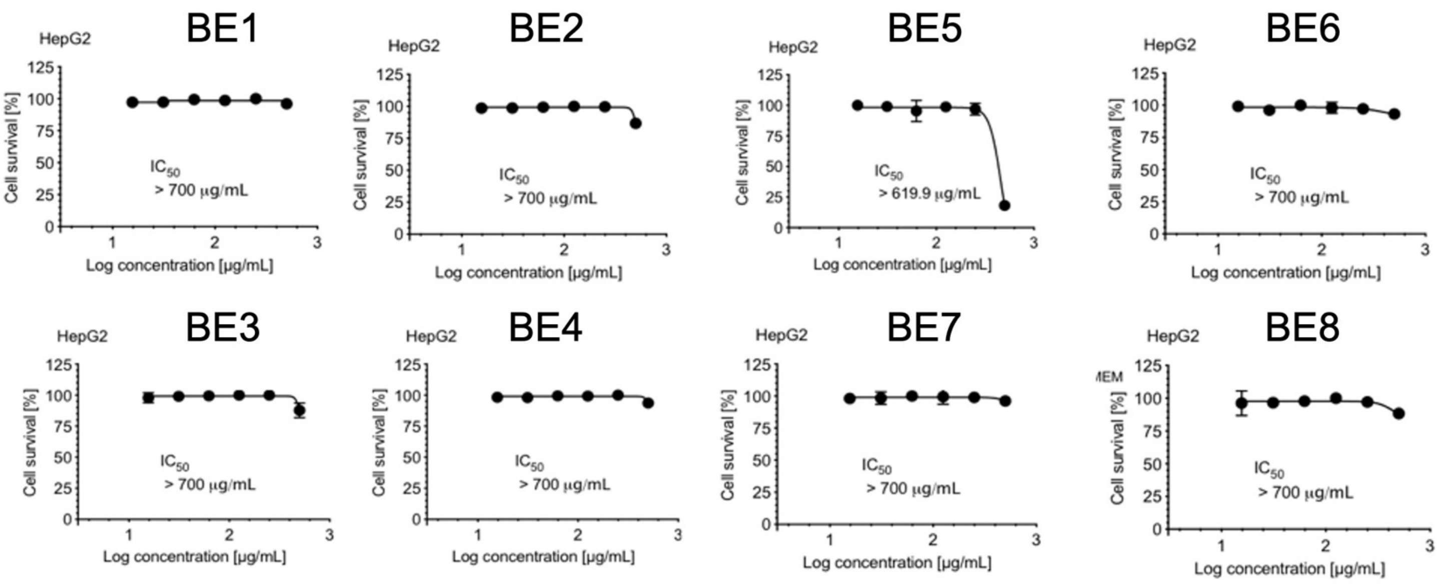

Figure S1. Cytotoxicity of selected bean samples in HepG2 cell lines

Table S2. Accumulation of TAG species induced by OA

Lipid species	RT	Ion	Calc. *m/z*	Exptl. *m/z*	ppm
TAG 42:0	25.32	$[M+NH_4]^+$	740.6763	740.6768	0.68
TAG 44:0	25.84	$[M+NH_4]^+$	768.7076	768.7078	0.26
TAG 46:1	25.92	$[M+NH_4]^+$	794.7232	794.7238	0.75
TAG 46:2	25.52	$[M+NH_4]^+$	792.7076	792.7073	-0.38
TAG 46:3	25.14	$[M+NH_4]^+$	790.6919	790.6915	-0.51
TAG 48:1	26.39	$[M+NH_4]^+$	822.7545	822.7559	1.70
TAG 48:2	26.03	$[M+NH_4]^+$	820.7389	820.7397	0.97
TAG 48:3	25.69	$[M+NH_4]^+$	818.7232	818.7233	0.12
TAG 48:4	25.40	$[M+NH_4]^+$	816.7076	816.7072	-0.49
TAG 50:2	26.53	$[M+NH_4]^+$	848.7702	848.7719	2.00
TAG 50:3	26.17	$[M+NH_4]^+$	846.7545	846.7550	0.59
TAG 50:4	25.87	$[M+NH_4]^+$	844.7389	844.7391	0.24
TAG 50:5	25.52	$[M+NH_4]^+$	842.7232	842.7233	0.12
TAG 50:6	25.32	$[M+NH_4]^+$	840.7076	840.7079	0.36
TAG 52:0	27.62	$[M+NH_4]^+$	880.8328	880.8284	-5.00
TAG 52:1	27.76	$[M+NH_4]^+$	878.8171	878.8152	-2.16
TAG 52:2	26.98	$[M+NH_4]^+$	876.8015	876.8032	1.94
TAG 52:3	26.66	$[M+NH_4]^+$	874.7858	874.7863	0.57
TAG 52:4	26.25	$[M+NH_4]^+$	872.7702	872.7695	-0.80
TAG 52:5	26.06	$[M+NH_4]^+$	870.7545	870.7543	-0.23
TAG 52:6	25.73	$[M+NH_4]^+$	868.7389	868.7394	0.58
**Lipid species**	**RT**	**Ion**	**Calc. *m/z***	**Exptl. *m/z***	**ppm**

TAG 52:7	25.43	$[M+NH_4]^+$	866.7232	866.7234	0.23
TAG 52:8	25.05	$[M+NH_4]^+$	864.7076	864.7079	0.35
TAG 54:0	25.69	$[M+NH_4]^+$	908.8641	908.8636	-0.55
TAG 54:1	28.42	$[M+NH_4]^+$	906.8484	906.8461	-2.54
TAG 54:2	27.94	$[M+NH_4]^+$	904.8328	904.8302	-2.87
TAG 54:3	27.47	$[M+NH_4]^+$	902.8171	902.8184	1.44
TAG 54:4	26.79	$[M+NH_4]^+$	900.8015	900.8013	-0.22
TAG 54:5	26.42	$[M+NH_4]^+$	898.7858	898.7861	0.33
TAG 54:6	26.20	$[M+NH_4]^+$	896.7702	896.7700	-0.22
TAG 54:7	25.92	$[M+NH_4]^+$	894.7545	894.7550	0.56
TAG 54:8	25.58	$[M+NH_4]^+$	892.7389	892.7394	0.56
TAG 54:9	25.32	$[M+NH_4]^+$	890.7232	890.7236	0.45
TAG 56:10	25.43	$[M+NH_4]^+$	916.7389	916.7401	1.31
TAG 56:4	27.62	$[M+NH_4]^+$	928.8328	928.8319	-0.97
TAG 56:5	26.93	$[M+NH_4]^+$	926.8171	926.8131	-4.32
TAG 56:6	26.77	$[M+NH_4]^+$	924.8015	924.8011	-0.43
TAG 56:7	26.34	$[M+NH_4]^+$	922.7858	922.7856	-0.22
TAG 56:8	26.06	$[M+NH_4]^+$	920.7702	920.7701	-0.11
TAG 56:9	25.73	$[M+NH_4]^+$	918.7545	918.7556	1.20
TAG 58:10	25.92	$[M+NH_4]^+$	944.7702	944.7706	0.42
TAG 58:11	25.63	$[M+NH_4]^+$	942.7545	942.7555	1.06
TAG 58:12	25.48	$[M+NH_4]^+$	940.7389	940.7436	5.00
TAG 58:13	27.94	$[M+NH_4]^+$	938.7232	938.7250	1.92
TAG 58:6	27.53	$[M+NH_4]^+$	952.8328	952.8324	-0.42
TAG 58:7	26.79	$[M+NH_4]^+$	950.8171	950.8135	-3.79
TAG 58:8	26.56	$[M+NH_4]^+$	948.8015	948.8010	-0.53
TAG 58:9	26.23	$[M+NH_4]^+$	946.7858	946.7856	-0.21

Lipid species	RT	Ion	Calc. *m/z*	Exptl. *m/z*	ppm
TAG 60:10	26.31	$[M+NH_4]^+$	972.8015	972.8010	-0.51
TAG 60:11	26.09	$[M+NH_4]^+$	970.7858	970.7859	0.10
TAG 60:12	25.84	$[M+NH_4]^+$	968.7702	968.7706	0.41
TAG 60:13	25.43	$[M+NH_4]^+$	966.7545	966.7545	0.00
TAG 62:12	26.23	$[M+NH_4]^+$	996.8015	996.8016	0.10
TAG 62:13	25.95	$[M+NH_4]^+$	994.7858	994.7866	0.80
TAG 62:14	25.66	$[M+NH_4]^+$	992.7702	992.7708	0.60
TAG 64:16	25.52	$[M+NH_4]^+$	1016.7702	1016.7735	3.25

Table S3. Accumulation of TGOOH species induced by OA

Lipid species	RT	Ion	Calc. *m/z*	Exptl. *m/z*	ppm
TG-OOH 46:2	21.84	$[M+NH_4]^+$	824.6974	824.6974	0.00
TG-OOH 48:1	26.98	$[M+NH_4]^+$	854.7443	854.7430	-1.52
TG-OOH 48:3	28.67	$[M+NH_4]^+$	850.7130	850.7172	4.94
TG-OOH 48:5	26.14	$[M+NH_4]^+$	846.6817	846.6844	3.19
TG-OOH 54:10	25.84	$[M+NH_4]^+$	920.6974	920.6976	0.22
TG-OOH 60:13	20.56	$[M+NH_4]^+$	998.7443	998.7397	-4.61
TG-OOH 60:14	20.29	$[M+NH_4]^+$	996.7287	996.7333	4.62
TG-OOH 60:15	19.62	$[M+NH_4]^+$	994.7130	994.7175	4.52

Table S4. Accumulation of TAG species induced by LA

Lipid species	RT	Ion	Calc. *m/z*	Exptl. *m/z*	ppm
TAG 42:0	13.42	$[M+NH_4]^+$	740.6763	740.6773	1.35
TAG 44:0	13.83	$[M+NH_4]^+$	768.7076	768.7083	0.91
TAG 46:0	14.32	$[M+NH_4]^+$	796.7389	796.7401	1.51
TAG 46:1	13.9	$[M+NH_4]^+$	794.7232	794.7234	0.25
TAG 46:2	13.59	$[M+NH_4]^+$	792.7076	792.7084	1.01
TAG 46:3	13.27	$[M+NH_4]^+$	790.6919	790.6924	0.63
TAG 48:0	14.69	$[M+NH_4]^+$	824.7702	824.7711	1.09
TAG 48:1	14.36	$[M+NH_4]^+$	822.7545	822.7560	1.82
TAG 48:2	14.05	$[M+NH_4]^+$	820.7389	820.7401	1.46
TAG 48:3	13.67	$[M+NH_4]^+$	818.7232	818.7238	0.73
TAG 48:4	13.39	$[M+NH_4]^+$	816.7076	816.7076	0.00
TAG 48:5	11.19	$[M+NH_4]^+$	814.6169	814.6196	3.31
TAG 50:0	15.09	$[M+NH_4]^+$	852.8015	852.8029	1.64
TAG 50:1	14.72	$[M+NH_4]^+$	850.7858	850.7872	1.65
TAG 50:2	14.43	$[M+NH_4]^+$	848.7702	848.7718	1.89
TAG 50:3	14.11	$[M+NH_4]^+$	846.7545	846.7560	1.77
TAG 50:4	13.79	$[M+NH_4]^+$	844.7389	844.7404	1.78
TAG 50:5	13.52	$[M+NH_4]^+$	842.7232	842.7241	1.07
TAG 50:6	13.25	$[M+NH_4]^+$	840.7076	840.7079	0.36
TAG 52:0	15.48	$[M+NH_4]^+$	880.8328	880.8345	1.93
**Lipid species**	**RT**	**Ion**	**Calc. *m/z***	**Exptl. *m/z***	**ppm**

**Lipid species**	**RT**	**Ion**	**Calc. *m/z***	**Exptl. *m/z***	**ppm**
TAG 52:1	15.1	$[M+NH_4]^+$	878.8171	878.8178	0.80
TAG 52:2	14.78	$[M+NH_4]^+$	876.8015	876.8026	1.25
TAG 52:3	14.5	$[M+NH_4]^+$	874.7858	874.7870	1.37
TAG 52:4	14.21	$[M+NH_4]^+$	872.7702	872.7720	2.06
TAG 52:5	13.87	$[M+NH_4]^+$	870.7545	870.7556	1.26
TAG 52:6	13.61	$[M+NH_4]^+$	868.7389	868.7401	1.38
TAG 52:7	13.42	$[M+NH_4]^+$	866.7232	866.7239	0.81
TAG 52:8	13.18	$[M+NH_4]^+$	864.7076	864.7079	0.35
TAG 54:0	15.89	$[M+NH_4]^+$	908.8641	908.8655	1.54
TAG 54:1	15.5	$[M+NH_4]^+$	906.8484	906.8481	-0.33
TAG 54:10	13.08	$[M+NH_4]^+$	888.7076	888.7095	2.14
TAG 54:2	15.16	$[M+NH_4]^+$	904.8328	904.8331	0.33
TAG 54:3	14.89	$[M+NH_4]^+$	902.8171	902.8172	0.11
TAG 54:4	14.61	$[M+NH_4]^+$	900.8015	900.8013	-0.22
TAG 54:5	14.3	$[M+NH_4]^+$	898.7858	898.7859	0.11
TAG 54:6	13.98	$[M+NH_4]^+$	896.7702	896.7712	1.12
TAG 54:7	13.72	$[M+NH_4]^+$	894.7545	894.7554	1.01
TAG 54:8	13.58	$[M+NH_4]^+$	892.7389	892.7389	0.00
TAG 54:9	13.33	$[M+NH_4]^+$	890.7232	890.7243	1.23
TAG 56:10	13.44	$[M+NH_4]^+$	916.7389	916.7397	0.87
TAG 56:11	13.27	$[M+NH_4]^+$	914.7232	914.7255	2.51
TAG 56:4	14.98	$[M+NH_4]^+$	928.8328	928.8322	-0.65
TAG 56:5	14.67	$[M+NH_4]^+$	926.8171	926.8161	-1.08

TAG 56:6	14.41	$[M+NH_4]^+$	924.8015	924.8007	-0.87
TAG 56:7	14.14	$[M+NH_4]^+$	922.7858	922.7849	-0.98
TAG 56:8	13.89	$[M+NH_4]^+$	920.7702	920.7695	-0.76
TAG 56:9	13.67	$[M+NH_4]^+$	918.7545	918.7542	-0.33
TAG 58:10	13.81	$[M+NH_4]^+$	944.7702	944.7708	0.64
TAG 58:11	13.59	$[M+NH_4]^+$	942.7545	942.7546	0.11
TAG 58:12	13.42	$[M+NH_4]^+$	940.7389	940.7426	3.93
TAG 58:13	12.18	$[M+NH_4]^+$	938.7232	938.7241	0.96
TAG 58:6	14.81	$[M+NH_4]^+$	952.8328	952.8323	-0.52
TAG 58:7	14.59	$[M+NH_4]^+$	950.8171	950.8170	-0.11
TAG 58:8	14.38	$[M+NH_4]^+$	948.8015	948.8018	0.32
TAG 58:9	14.11	$[M+NH_4]^+$	946.7858	946.7856	-0.21
TAG 60:10	14.19	$[M+NH_4]^+$	972.8015	972.8018	0.31
TAG 60:11	14.03	$[M+NH_4]^+$	970.7858	970.7860	0.21
TAG 60:12	13.76	$[M+NH_4]^+$	968.7702	968.7706	0.41
TAG 60:13	13.52	$[M+NH_4]^+$	966.7545	966.7559	1.45
TAG 62:12	14.14	$[M+NH_4]^+$	996.8015	996.8019	0.40
TAG 62:13	13.83	$[M+NH_4]^+$	994.7858	994.7877	1.91
TAG 62:14	13.67	$[M+NH_4]^+$	992.7702	992.7711	0.91
TAG 64:16	13.52	$[M+NH_4]^+$	1016.7702	1016.7745	4.23

Table S5. Accumulation of TGOOH species induced by OA

Lipid species	RT	Ion	Calc. *m/z*	Exptl. *m/z*	Ppm
TG-OOH 48:1	9.17	$[M+NH_4]^+$	854.7443	854.7481	4.45
TG-OOH 52:2	12.59	$[M+NH_4]^+$	908.7913	908.7930	1.87
TG-OOH 54:7	13.61	$[M+NH_4]^+$	926.7443	926.7442	-0.11
TG-OOH 56:10	10.13	$[M+NH_4]^+$	948.7287	948.7265	-2.32
TG-OOH 56:4	14.19	$[M+NH_4]^+$	960.8226	960.8207	-1.98
TG-OOH 56:5	13.81	$[M+NH_4]^+$	958.8069	958.8085	1.67
TG-OOH 58:10	10.79	$[M+NH_4]^+$	976.7600	976.7574	-2.66
TG-OOH 58:11	10.15	$[M+NH_4]^+$	974.7443	974.7418	-2.56
TG-OOH 58:6	13.98	$[M+NH_4]^+$	984.8226	984.8235	0.91
TG-OOH 58:7	13.7	$[M+NH_4]^+$	982.8069	982.8095	2.65
TG-OOH 58:8	13.44	$[M+NH_4]^+$	980.7913	980.7941	2.85
TG-OOH 60:11	13.79	$[M+NH_4]^+$	1002.7756	1002.7768	1.20
TG-OOH 60:13	10.79	$[M+NH_4]^+$	998.7443	998.7402	-4.11
TG-OOH 60:14	10.75	$[M+NH_4]^+$	996.7287	996.7336	4.92
TG-OOH 60:15	10.15	$[M+NH_4]^+$	994.7130	994.7174	4.42

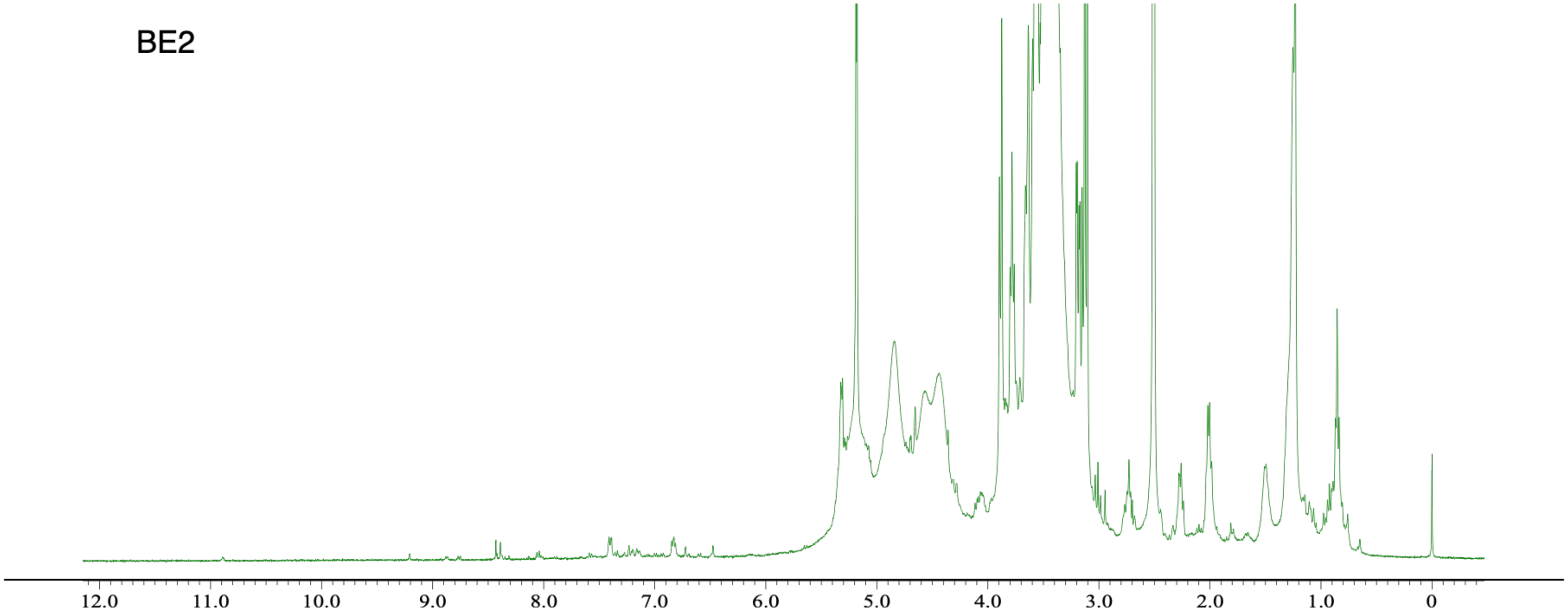

Figure S2. ^{1}H NMR spectrum of BE2 in DMSO-d6

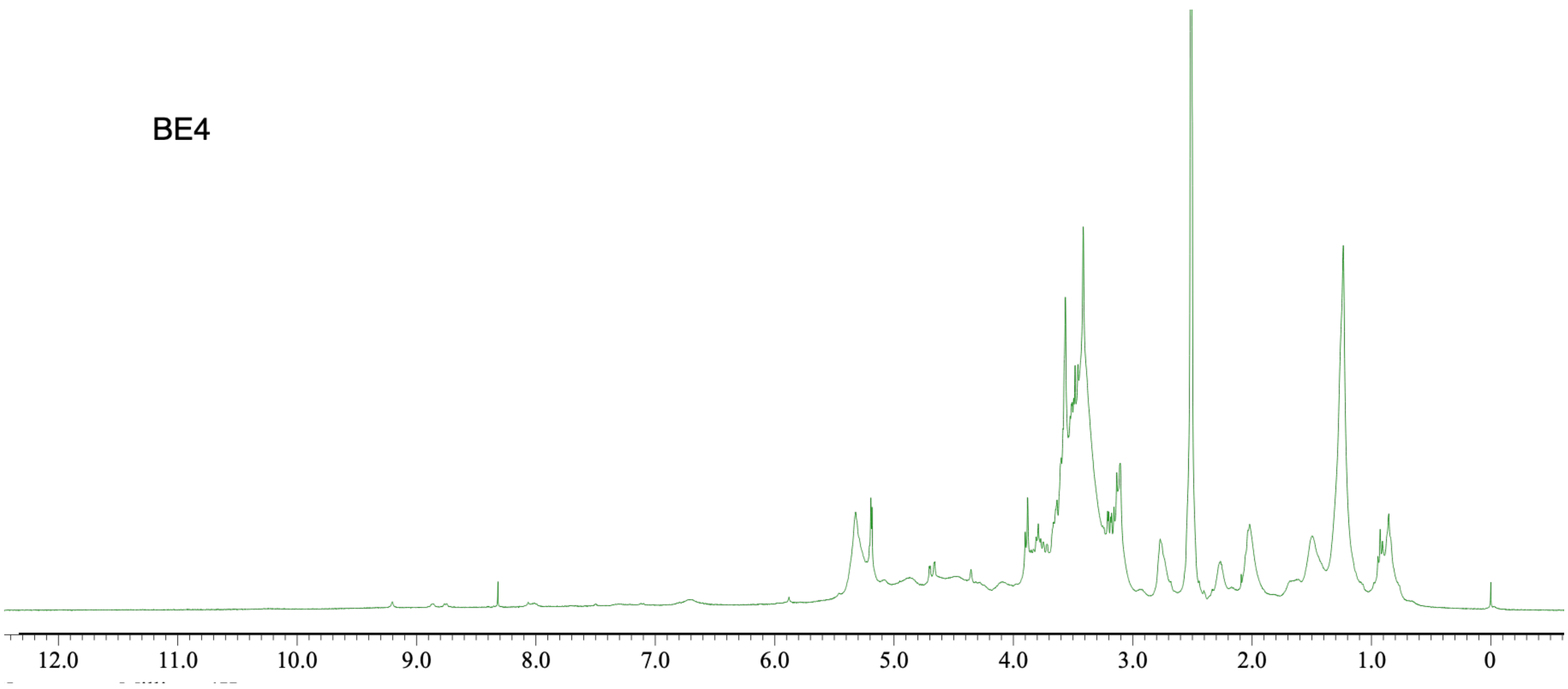

Figure S3. ^{1}H NMR spectrum of BE4 in DMSO-d6

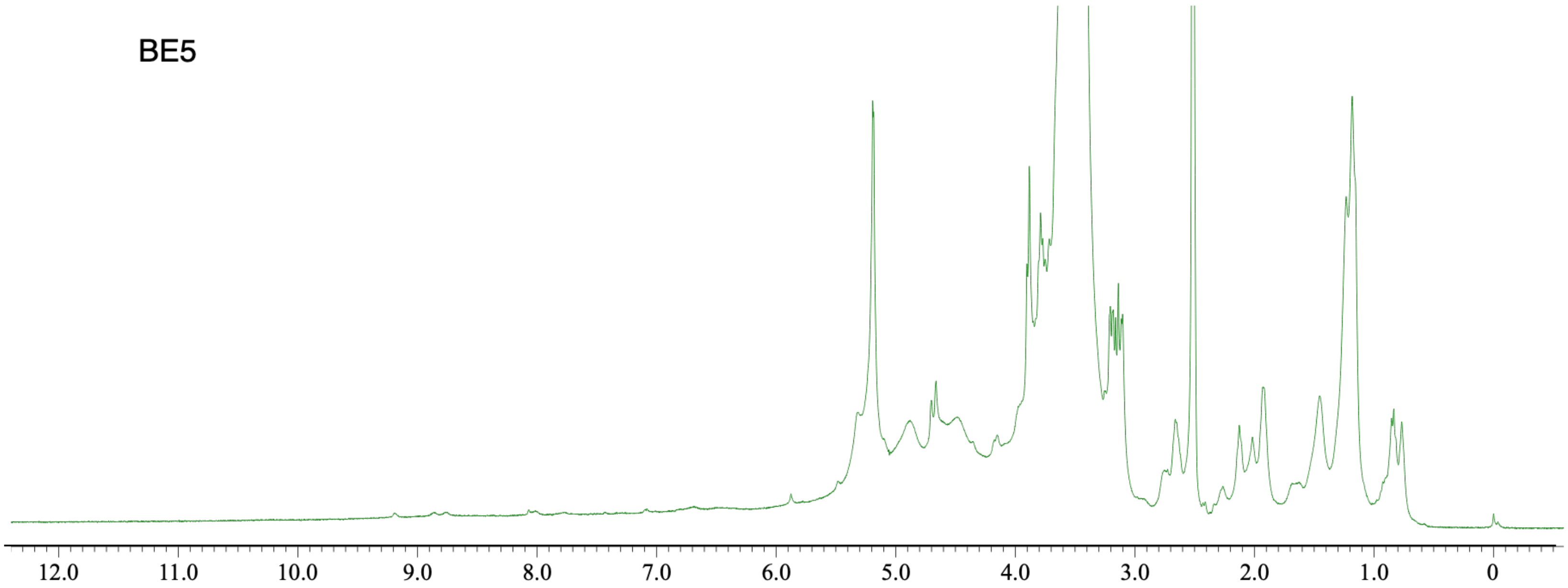

Figure S4. ^{1}H NMR spectrum of BE5 in DMSO-d6

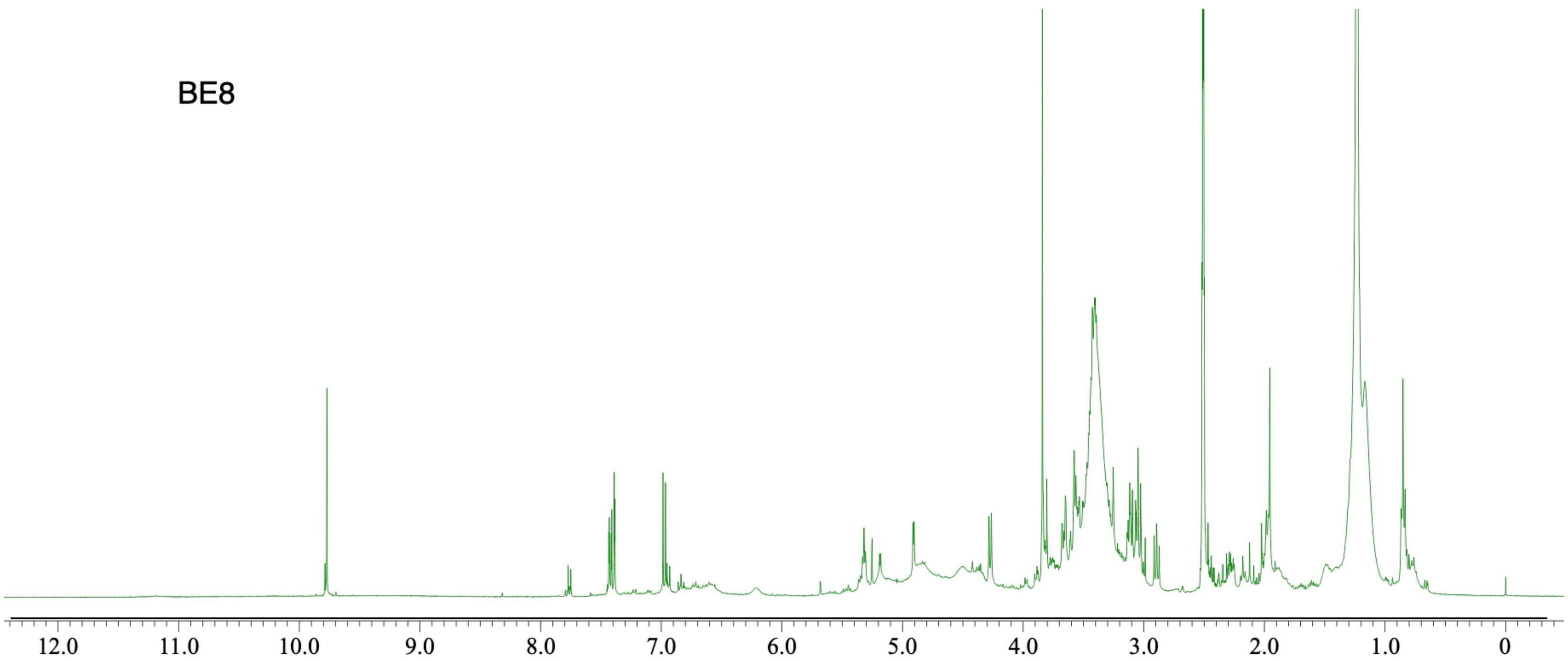

Figure S5. ^{1}H NMR spectrum of BE8 in DMSO-d6

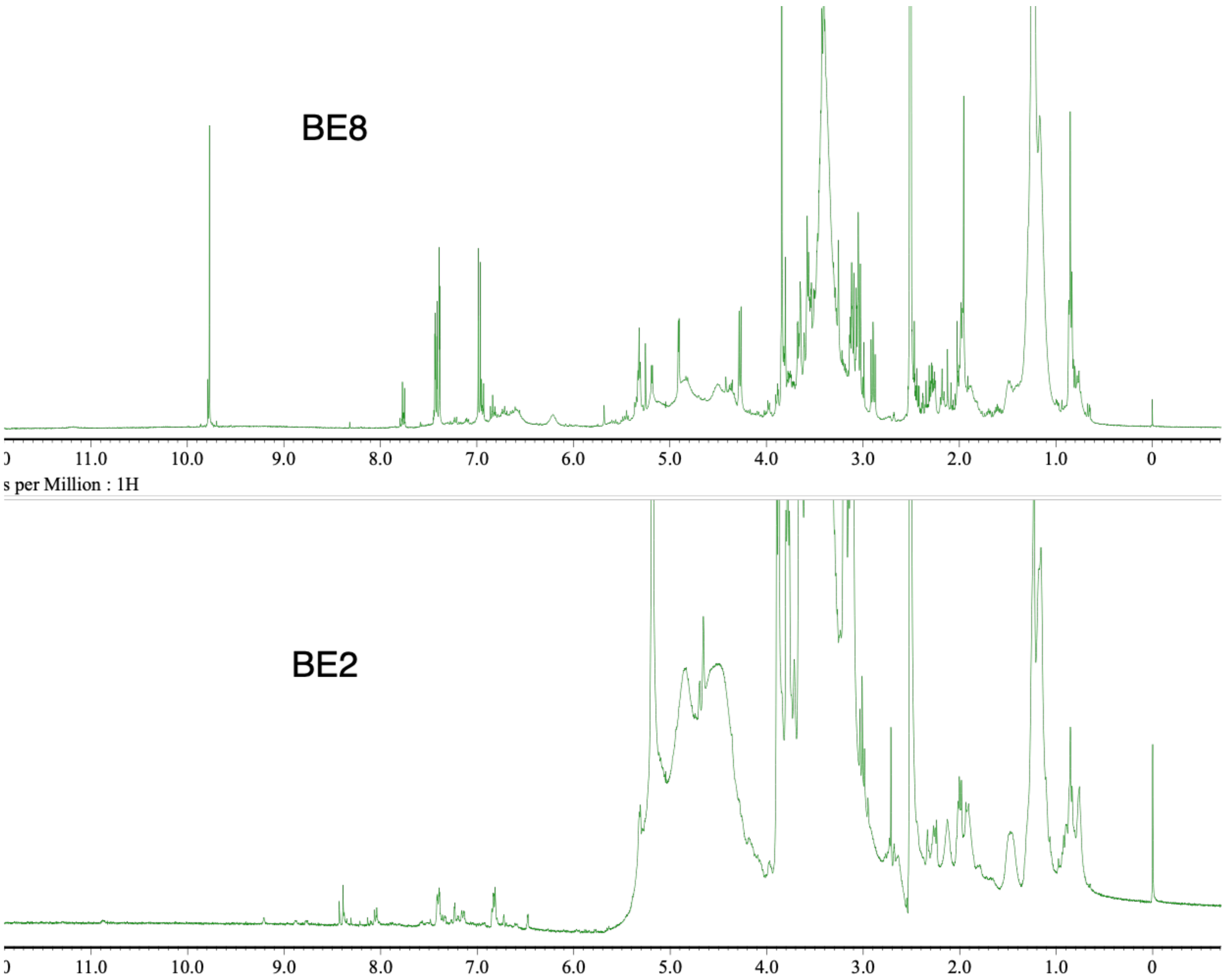

Figure S6. ^{1}H NMR spectrum of bioactive extract BEs (BE2 and BE8) in DMSO-d6

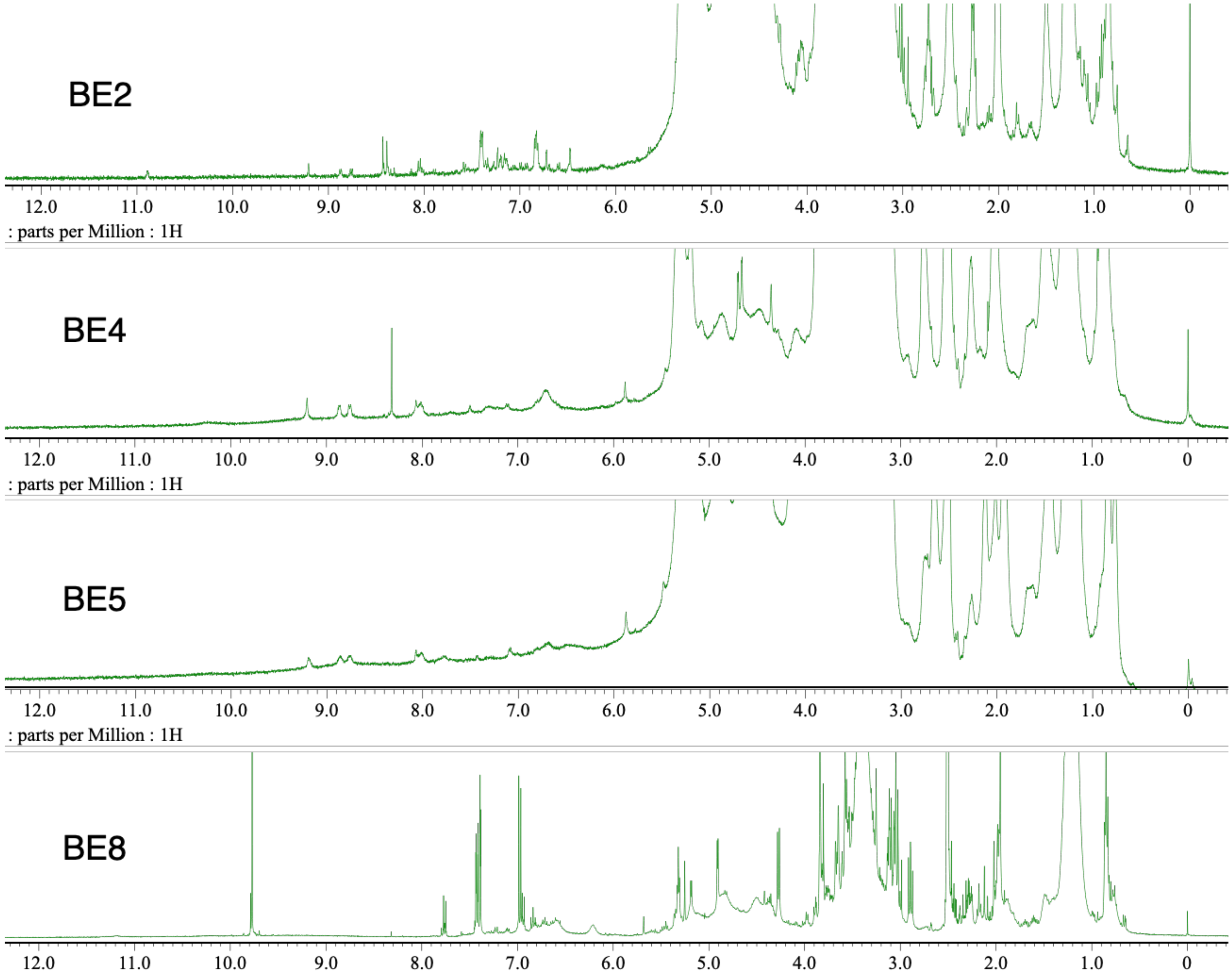

Figure S7. ^{1}H NMR spectrum of bioactive extract BEs (BE2 and BE8) in DMSO-d6

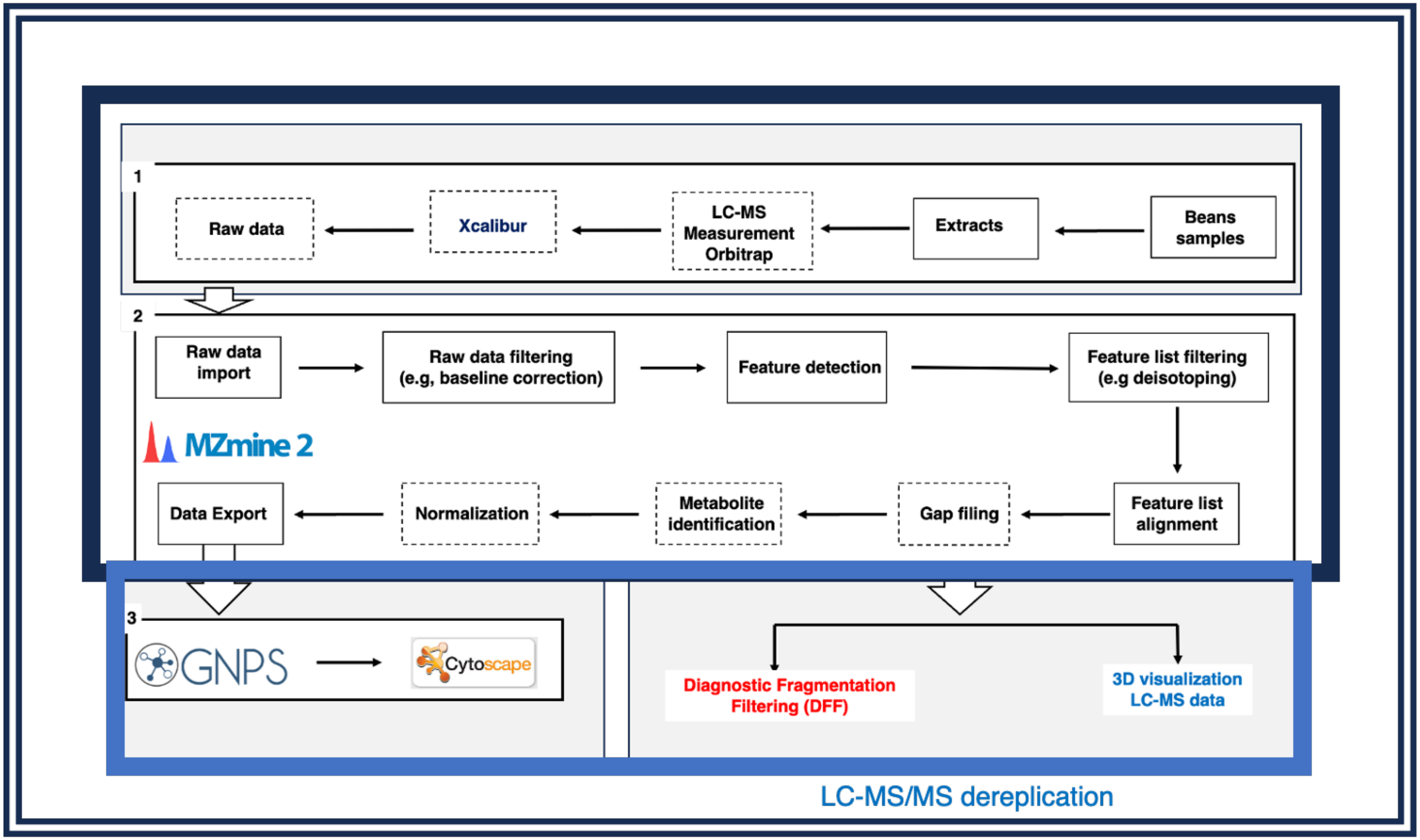

Figure S8. A schema of the general data processing workflow of LC-MS data

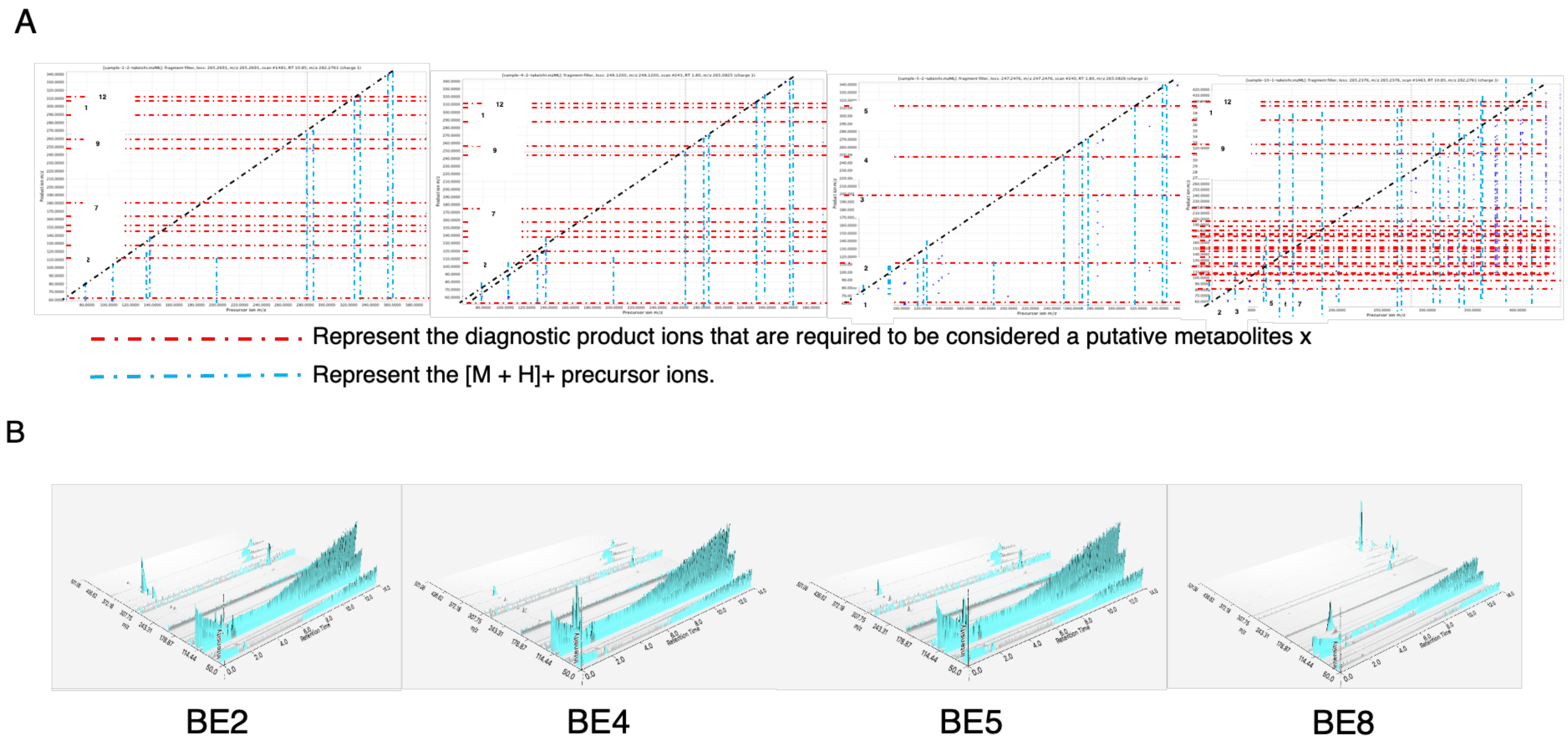

Figure S9. LC-MS profiling of bioactive bean extracts. (A) Diagnostic Fragmentation Filtering (DFF) plot for metabolites analysis. (B) 3D visualization of MS data.

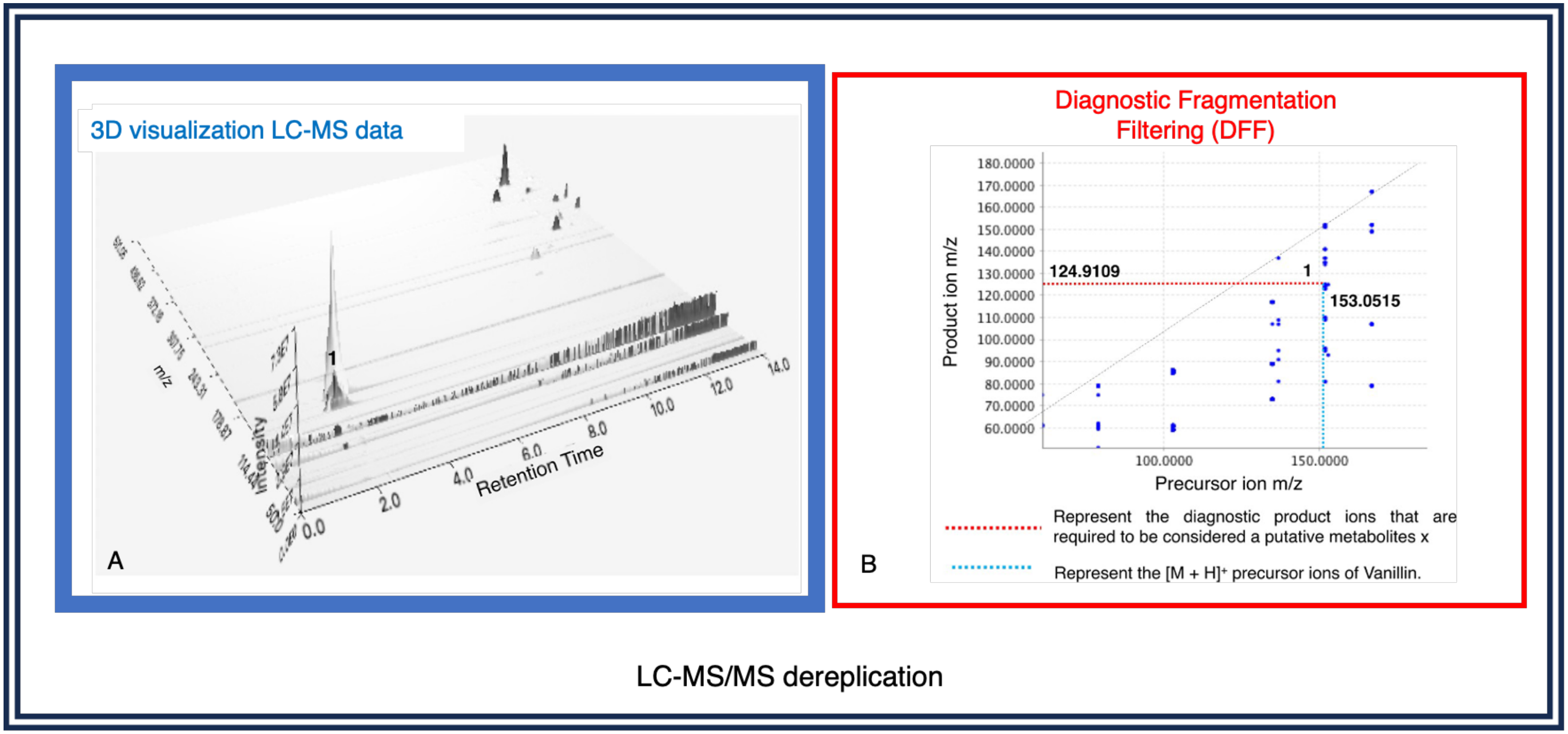

Figure S10. Identification of vanillin in BE8. (A) Three-dimensional (3D) liquid chromatography/mass spectrometry (LC-MS) of BE8 and vanillin. (B) Plot of the vanillin in BE8 using diagnostic fragmentation filtering (DFF).

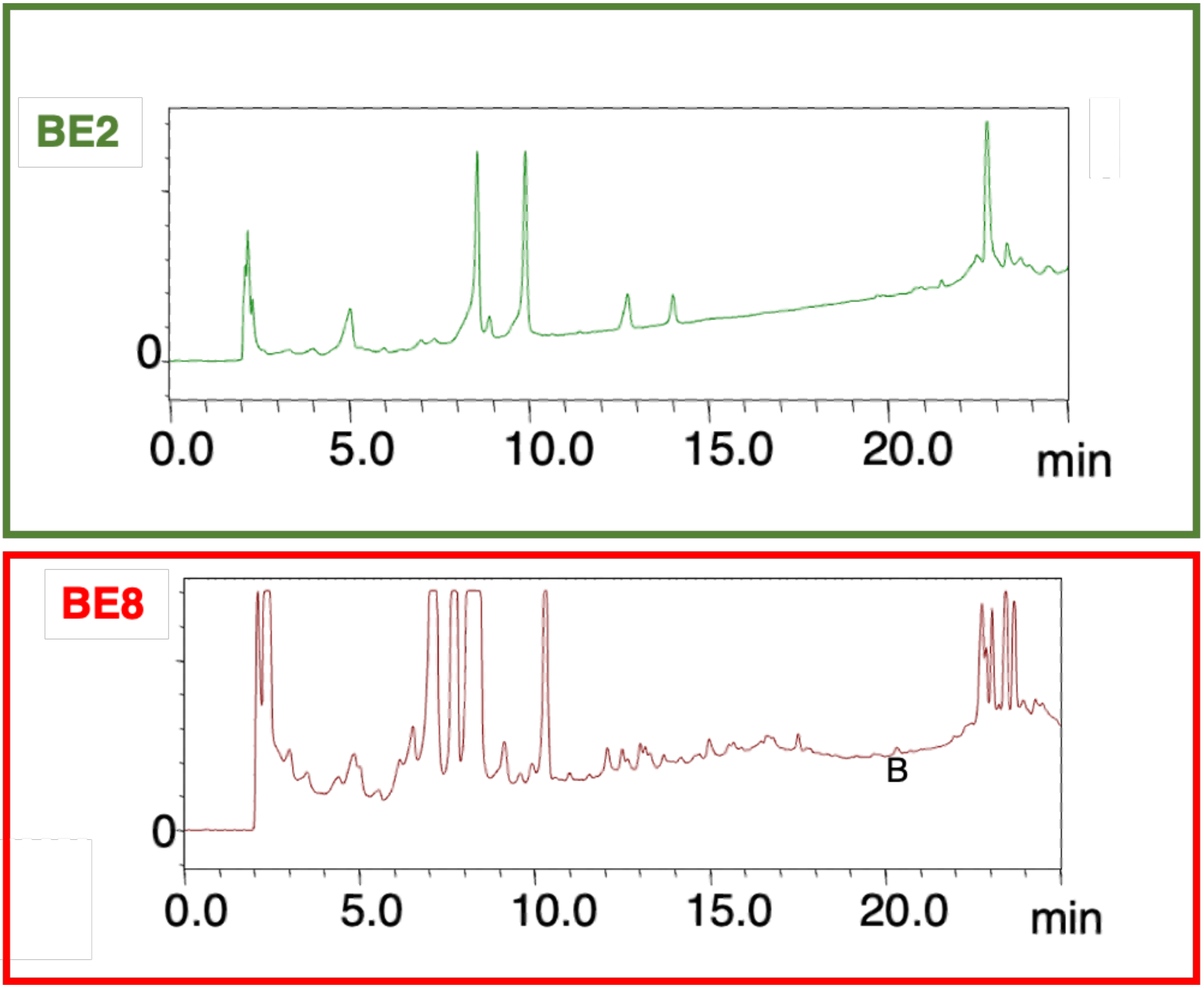

Figure S11. HPLC profile of bioactive bean BE2 and BE8 at 200nm

Supporting LDAI Imaging

Table of contents P25

1. **Scheme S1**. A. Capture of the LDA real time images on HepG2 cells with the interval of 6h. B. Vial ability staining with AO and EB. C. Evaluation of LDA and oxLDs inhibition strategy P26

2. **Scheme S2**. A. Comparison of the capture of the LDA under -OA and +OA in real time images on HepG2 cells with the interval of 12h. B. LDAI activity of BE1-BE8. C. Comparison of the vial ability staining with AO and EB under -OA and +OA P27

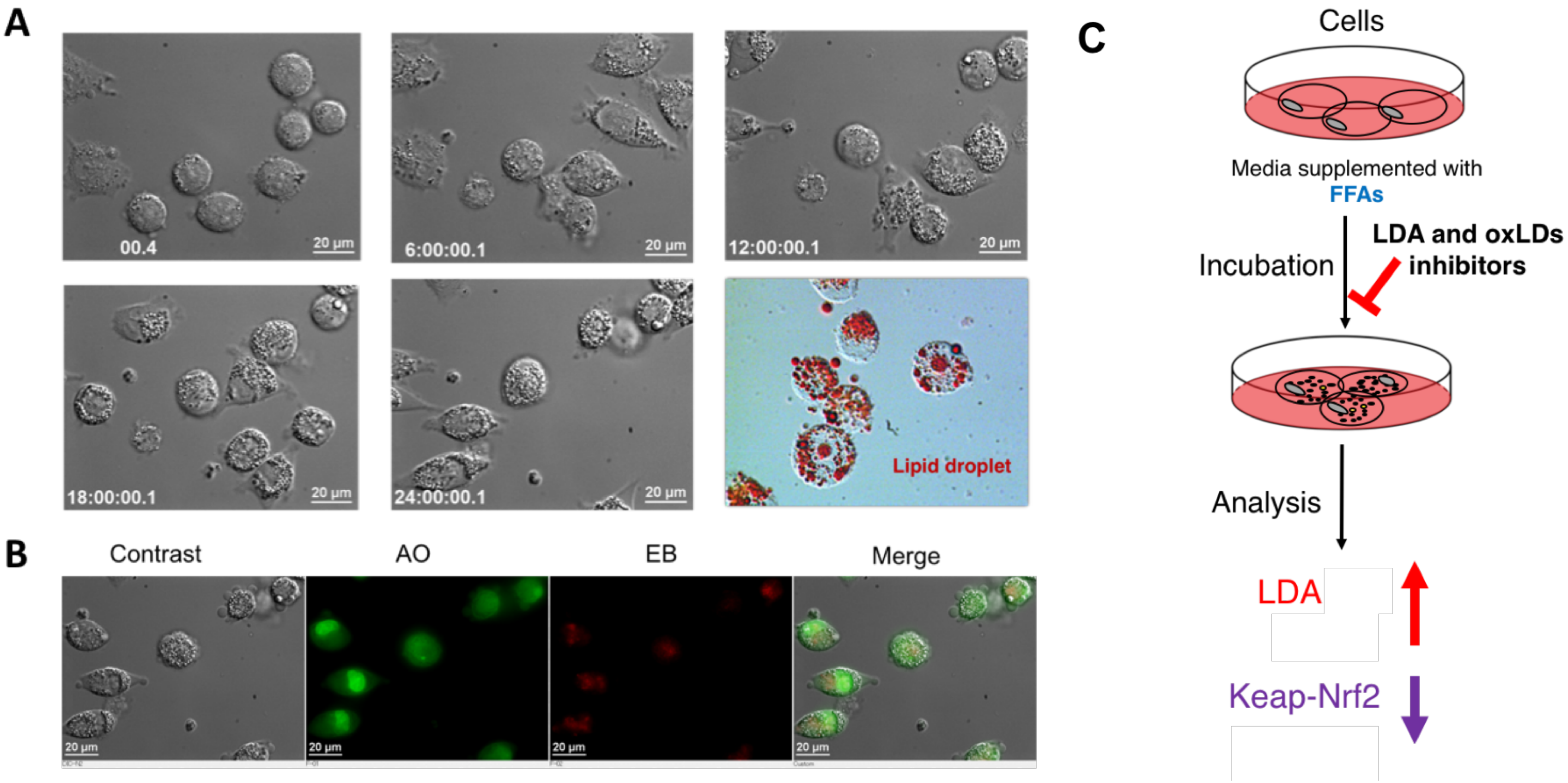

Scheme S1. A. Capture of the LDA real time images on HepG2 cells with theinterval of 6h. B Vialability staining with AO and EB. C. Evaluation of LDA and oxLDs inhibition strategy

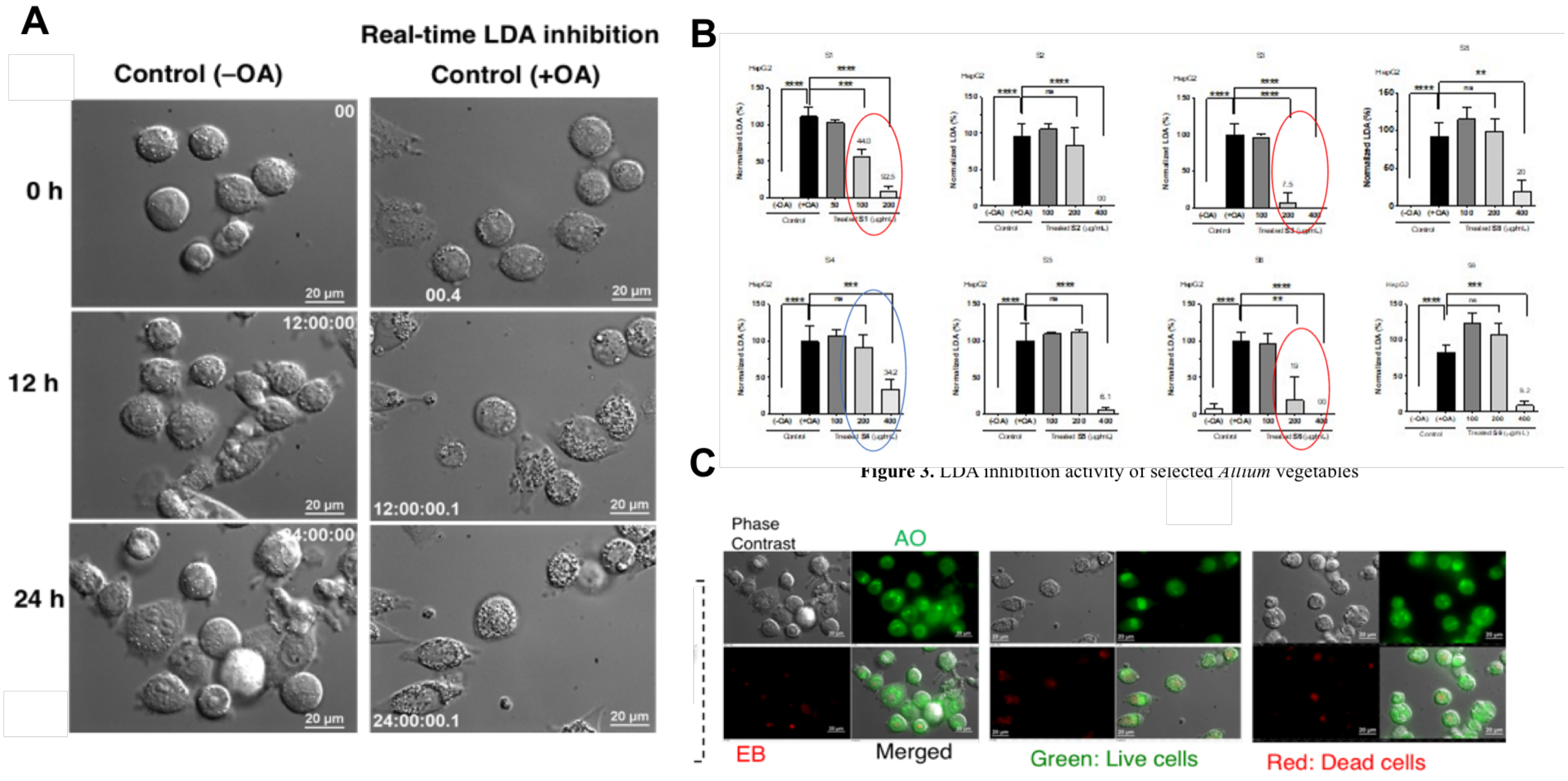

Scheme S2. A. Comparison of the capture of the LDA under -OA and +OA in real time images on HepG2 cells with the interval of 12h. B. LDAI activity of BE1-BE8. C. Comparison of the vial ability staining with AO and EB under –OA and +OA**.**
